# Acute anxiety during the COVID-19 pandemic was associated with higher levels of everyday altruism

**DOI:** 10.1038/s41598-022-23415-2

**Published:** 2022-11-03

**Authors:** Joana B. Vieira, Stephen Pierzchajlo, Simon Jangard, Abigail A. Marsh, Andreas Olsson

**Affiliations:** 1 Department of Psychology, Faculty of Health and Life Sciences, University of Exeter, Exeter, UK; 2 Department of Clinical Neuroscience, Karolinska Institutet, Stockholm, Sweden; 3 Department of Psychology, Stockholm University, Stockholm, Sweden; 4 Department of Psychology, Georgetown University, Washington, DC USA

**Keywords:** Psychology, Human behaviour

## Abstract

Prior laboratory research has suggested that humans may become more prosocial in stressful or threatening situations, but it is unknown whether the link between prosociality and defense generalizes to real-life. Here, we examined the association between defensive responses to a real-world threat (the COVID-19 pandemic) and everyday altruism. Four independent samples of 150 (N = 600) US residents were recruited online at 4 different timepoints, and self-report measures of perceived COVID-19 threat, defensive emotions (e.g., stress and anxiety), and everyday altruism were collected. Our operationalization of defensive emotions was inspired by the threat imminence framework, an ecological model of how humans and animals respond to varying levels of threat. We found that perceived COVID-19 threat was associated with higher levels of everyday altruism (assessed by the Self-report Altruism scale). Importantly, there was a robust association between experiencing acute anxiety and high physiological arousal during the pandemic (responses typically characteristic of higher perceived threat imminence), and propensity to engage in everyday altruism. Non-significant or negative associations were found with less acute defensive responses like stress. These findings support a real-life relation between defensive and altruistic motivation in humans, which may be modulated by perceived threat imminence.

## Introduction

The beginning of 2020 was met with an unprecedented global challenge—the novel coronavirus outbreak. A defining feature of the COVID-19 pandemic, especially its earlier stages, was a widespread feeling of being under an invisible threat, a feature likely to profoundly impact psychological functioning and behaviour. Indeed, a wealth of laboratory research has demonstrated how defensive processes triggered by stressful or threatening situations affect not only psychological health^1^ and decision-making^2^, but also prosocial behaviour^3^. Still, little is known about how ecological threats of varying proximity impact behaviour outside the laboratory setting. The pandemic presented a unique opportunity to answer this question. Here, we examined the association between changing COVID-19 threat and prosocial behaviour, particularly everyday altruism.

There is a long held popular view that human nature is inherently self-serving, and presumably more likely to reveal itself in challenging contexts or when resources are scarce^4^. These characteristics are applicable to early stages of a pandemic, wherein the fear of infection co-occurred with that of losing one's job and/or access to essential goods like medication, food, or even toilet paper. However, the notion of a fundamentally selfish human nature is called into question by the ubiquitous nature of everyday altruism in modern societies^5,6^, and by instances of extraordinary altruism in highly risky (e.g., heroic rescues; https://www.carnegiehero.org/) or costly scenarios (e.g., non-directed organ donation)^7^. Importantly, it has been suggested that challenging contexts may in fact promote rather than hinder altruistic motivation^3,8^. Previous studies have shown that inducing acute social stress through paradigms like the Trier Social Stress Test increases prosocial behaviour in subsequent economic exchanges^9^ and hypothetical moral decisions^10^, as well as the ability to empathize with others' pain^11^. Other studies, however, indicated the link between stress and prosocial behaviour is more nuanced, as it may be shaped by additional individual and situational variables^12–14^.

One such variable could be the imminence of danger, and the way it is subjectively perceived by individuals. It has been proposed that, in humans and other species, defensive responses vary based on the spatiotemporal proximity of the threat, along a so-called threat imminence continuum^15^. Distal threats predominantly trigger vigilance, risk assessment and more flexible escape strategies, whereas imminent threats activate more stereotyped avoidance responses (e.g., freezing and fight-or-flight)^15–17^. The terms pre-encounter, post-encounter, and circa-strike have been used to describe different defensive contexts along the imminence continuum, and research in both animals and humans has begun to elucidate the neural circuitries implicated in adaptively responding to each context (see^17^ for a review). Importantly, it has been proposed that human behaviours, cognitions and emotions that characterize states like anxiety, fear and panic may be understood as contiguous transitions in response to the perceived imminence of a threat^17–19^. For example, intermittent anticipatory anxiety accompanied by cognitive control and reappraisal strategies are typical in response to an unpredictable threat that is not immediately present but may appear at any point (pre-encounter). By contrast, encounter anxiety may result from a threat that is already present but has consequences that are still somewhat unpredictable (post-encounter), and acute fear or panic may arise from a threat that is present and predictable, for which immediate avoidance is needed (circa-strike). An important point is that defensive responses are known to be variable between individuals^20–22^, and it is thus reasonable to expect some degree of inter-individual variability in this defensive continuum, i.e., a threat may be perceived as more imminent by some than others, giving rise to varying defensive behaviours to the same external event.

There is laboratory evidence that defensive states along the imminence continuum may have different effects on prosocial behaviour. Specifically, we have recently demonstrated that healthy individuals were more likely to help a co-participant avoid aversive electrical shocks (at the risk of also being shocked) when helping decisions were made immediately before the shock delivery (imminent threat) than in the beginning of the trial (distal threat)^23^. Further, responses made during imminent relative to distal threats were faster, and accompanied by increased heart rate. These findings suggested that acute defensive states triggered in situations of imminent danger not only enable self-preservation responses, but may also promote motivation to defend/help others^24^. It is, however, unknown whether the link between threat imminence and altruistic motivation would persist in a larger spatiotemporal scale and in a real-life context. The COVID-19 pandemic offered the opportunity to examine this question. In the United States, the first confirmed case of COVID-19 was reported on January 21st, and the government declared the outbreak a Public Health Emergency on February 3rd. On March 11th, the World Health Organization officially classified the outbreak as a global pandemic. By then, other countries like China, Italy and Iran had already recorded a rampant number of cases. This sequence of unfolding events presumably contributed to a perception of increased threat imminence, as individuals witnessed the negative impact of the virus in other countries, and anticipated similar consequences in their own country.

Our main goal here was to determine the association between COVID-19 threat and everyday altruism, defined as voluntary actions that benefit others at a variable personal cost (e.g., money, time, effort). Based on our findings that increased threat imminence favors helping decisions^23^, and evidence that acute stress promotes prosociality^3,9–11^, we hypothesized that increased COVID-19 threat over time would be accompanied by an increase in self-reported altruistic behaviours at the population level. To test this, we performed four independent data collections (cross-sectional design), corresponding to one-week apart time points during the COVID-19 pandemic (March–April), during which number of confirmed cases and COVID-19 fatalities were objectively increasing. At each time point, self-report measures of everyday altruism, perceived COVID-19 threat, and experienced defensive emotional states were collected (see “Methods”). We then examined whether perceived COVID-19 threat and everyday altruism increased over those 4 weeks. Given the expected inter-individual variability in responding to threat imminence, we also hypothesized that individuals experiencing more acute defensive states (presumably linked with perception of higher threat imminence) would report higher engagement in everyday altruism. This prediction was tested by modelling everyday altruism as a function of different defensive emotions, while accounting for week-by-week variation. Our measure of everyday altruism was the Self-report Altruism Scale^26^, which assesses the frequency with which participants have engaged in different everyday altruistic behaviours in their lifetime. These ratings are believed to reflect both the frequency of performed acts, and the individual’s endorsement of helping others, thus being considered both a measure of self-reported behaviour and dispositional altruism^25^. This is an important point to keep in mind when interpreting our results, as they can reflect both behavioural and trait-level associations between defence and altruism.

## Results

### Changes in everyday altruism and perceived COVID-19 threat over time

**Figure 1:**
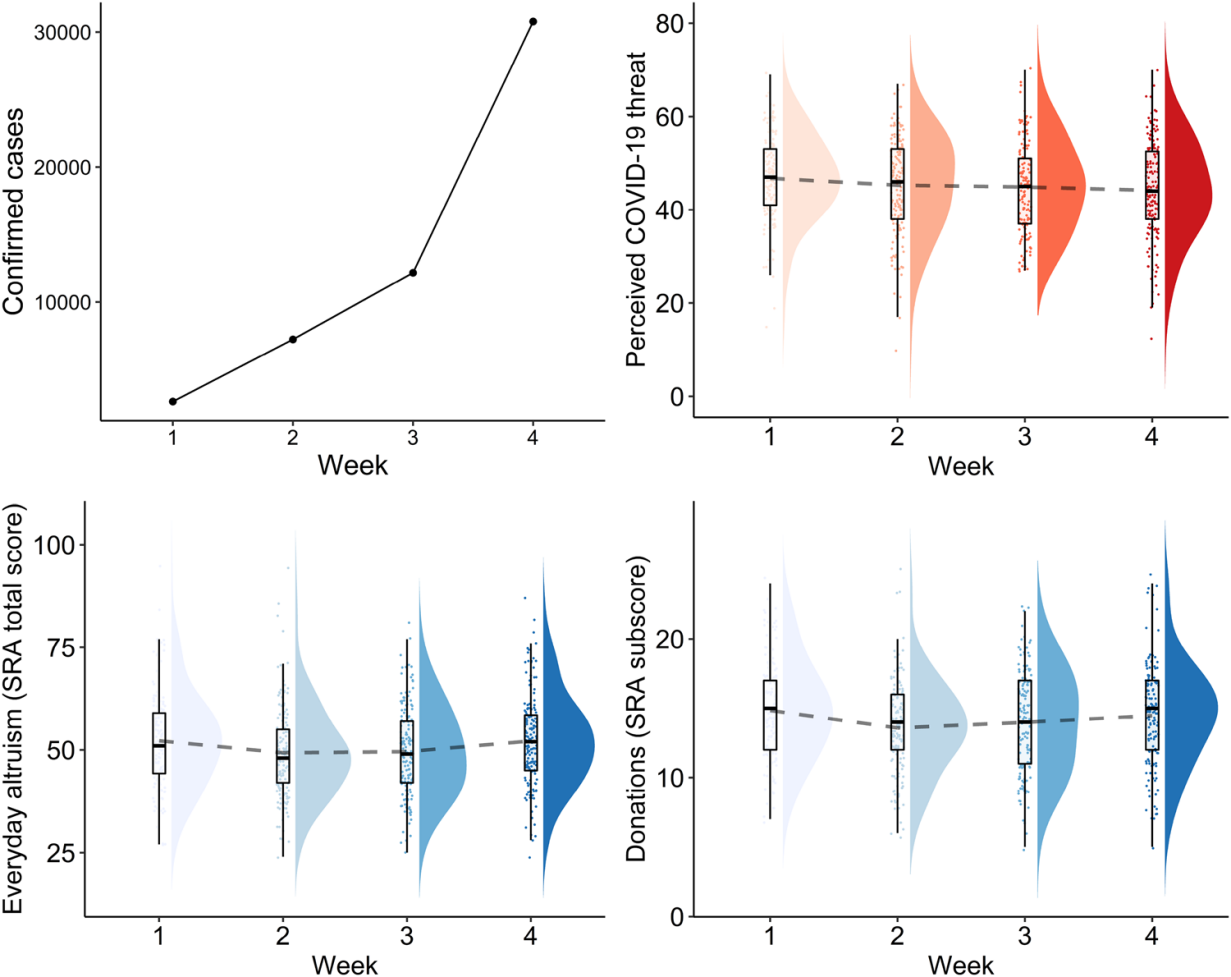
Total number of confirmed cases averaged across states, over the 4 weeks of data collection (top left); Perceived COVID-19 threat over the 4 weeks of data collection (top right ); Everyday altruism over the 4 weeks of data collection (SRA total and donations only score, bottom panel). The dashed line connects the mean across samples (i.e. weeks). Note that the distributions shown here correspond to independent samples collected on each week.

The total number of confirmed COVID-19 cases increased over the 4 weeks of data collection, but we found no evidence that perceived COVID-19 threat increased over time (*F*(3, 596) = 1.82, *p* = 0.14; Fig. 1). Regarding levels of everyday altruism assessed by the SRA, although the ANOVA suggested changes across the four weeks (*F*(3, 596 = 3.04, *p* = 0.03), follow-up pairwise comparisons were not statistically significant (all ps > 0.15).

Because some of the items of the SRA describe actions that may be difficult to perform during a lockdown (e.g., helping a stranger with car troubles), we repeated the ANOVA using as dependent variable a subset of SRA items referring to donations, given that the ability to carry out those behaviours should be less affected. When using the SRA-donations score, results suggested changes over the 4 weeks (*F*(3, 596) = 3.69, *p* = 0.012). Pairwise comparisons revealed the only significant difference was a decrease between Week 1 and 2 (*p* = 0.012).

Altogether, our results provided limited evidence about how population levels of overall altruism changed over the 4 weeks of data collection, but we found some indication that donation behaviour may have decreased from the first to the second week. These results could have been impacted by the non-changing threat perception, and/or limitations in the SRA’s ability to capture behavioural changes in that time period.

### Modelling everyday altruism (SRA) as a function of perceived threat and defensive emotions

To examine whether experiencing different defensive emotional states during the pandemic was associated with altruism at the individual level, we adopted a Generalized Linear Mixed Models (GLMMs) approach. This allowed us to account for variation in everyday altruism that was explained by our variables of interest (fixed effects), as well as by random sampling of timepoint (random effect) (see “Methods” and Supplementary material for a detail description of all the modelling checks and steps).

We first examined how everyday altruism (SRA) varied as a function of our key fixed effects of interest (Model 1), namely:

- Perceived COVID-19 threat (assessed by the COVID-19 Risk Perception Scale, RP^26^);
- Stress, defined as feelings of uncontrollability and unpredictability, which are characteristic in response to situations of lower threat imminence (assessed by Perceived Stress Scale-10, PSS-10^27^);
- Anxiety, defined as a state of high autonomic arousal, acute anxiety and panic, consistent with emotional responses to threats perceived as predictable and imminent (assessed by the Depression Anxiety Stress Scales, DASS-21^28^).

**Figure 2:**
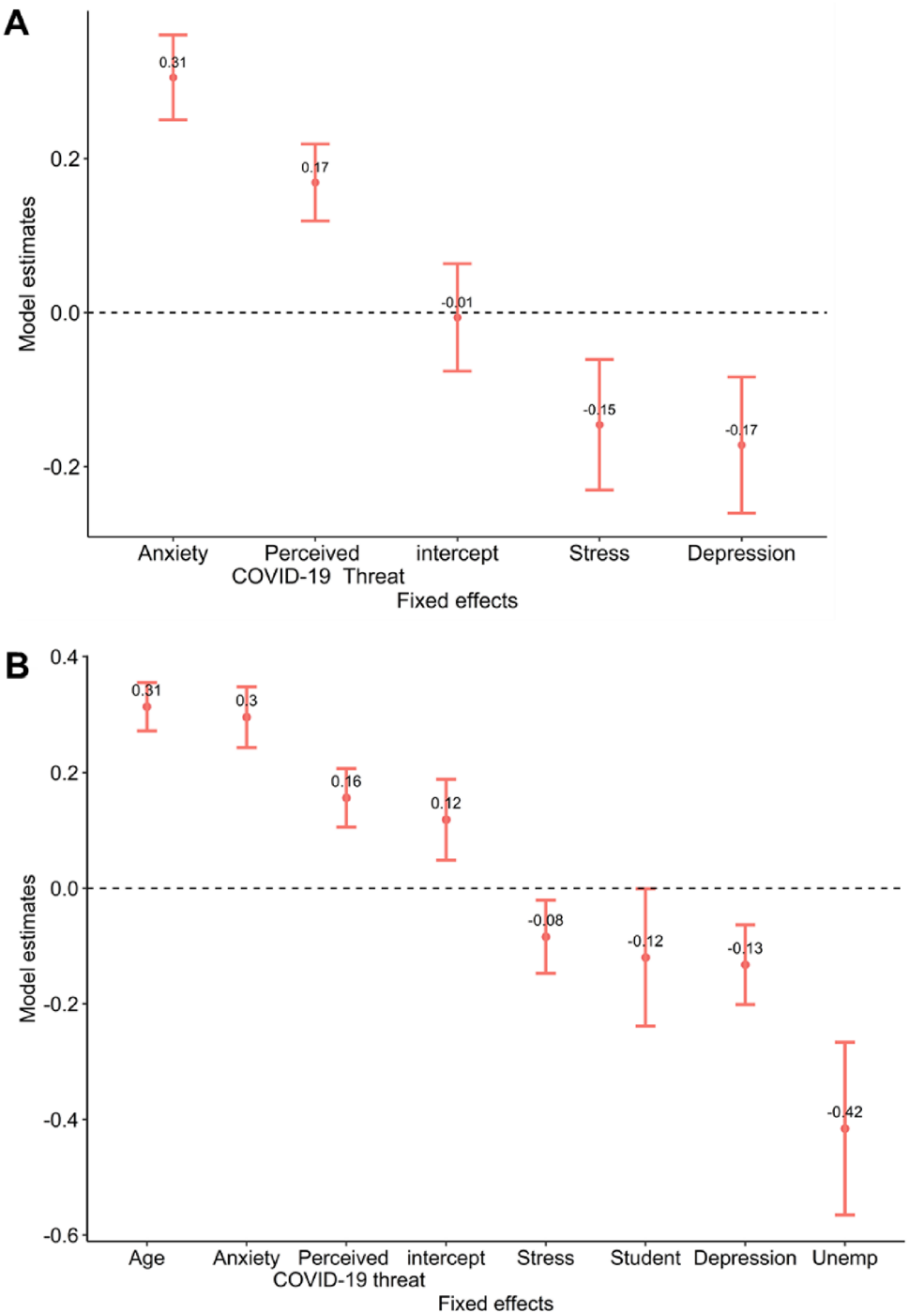
(**A**) Beta values and SEs for Model 1. (**B**) Beta values and SEs for Model 2. Dependent variable in both models is everyday altruism (SRA).

Although not the focus of our hypotheses, Depression (assessed by the DASS-21) was also included as a fixed effect, since it is thought to have important associations with stress and anxiety-related responses^29^. Results showed only perceived COVID-19 threat and anxiety were significantly associated with increased everyday altruism (Table 1, Fig. 2A). When modelling these variables with only random intercept per week, we found additionally that stress and depression were negatively associated with altruism (see Supplementary material—Addressing model singularity, Table m1.1).

**Table 1:** Model 1 estimates (DV: Everyday altruism, SRA).

	Estimate	Std. Error	df	t value	Pr(>|t|)	CI_lower	CI_upper
(Intercept)	− 0.006	0.070	3.084	− 0.091	0.933	− 0.143	0.131
Perceived COVID-19 threat	0.169	0.050	5.565	3.377	0.017*	0.071	0.267
Stress	− 0.146	0.085	3.282	− 1.719	0.176	− 0.312	0.020
Anxiety	0.305	0.055	9.893	5.526	0.000*	0.197	0.414
Depression	− 0.172	0.088	3.570	− 1.945	0.132	− 0.345	0.001

**Figure 3:**
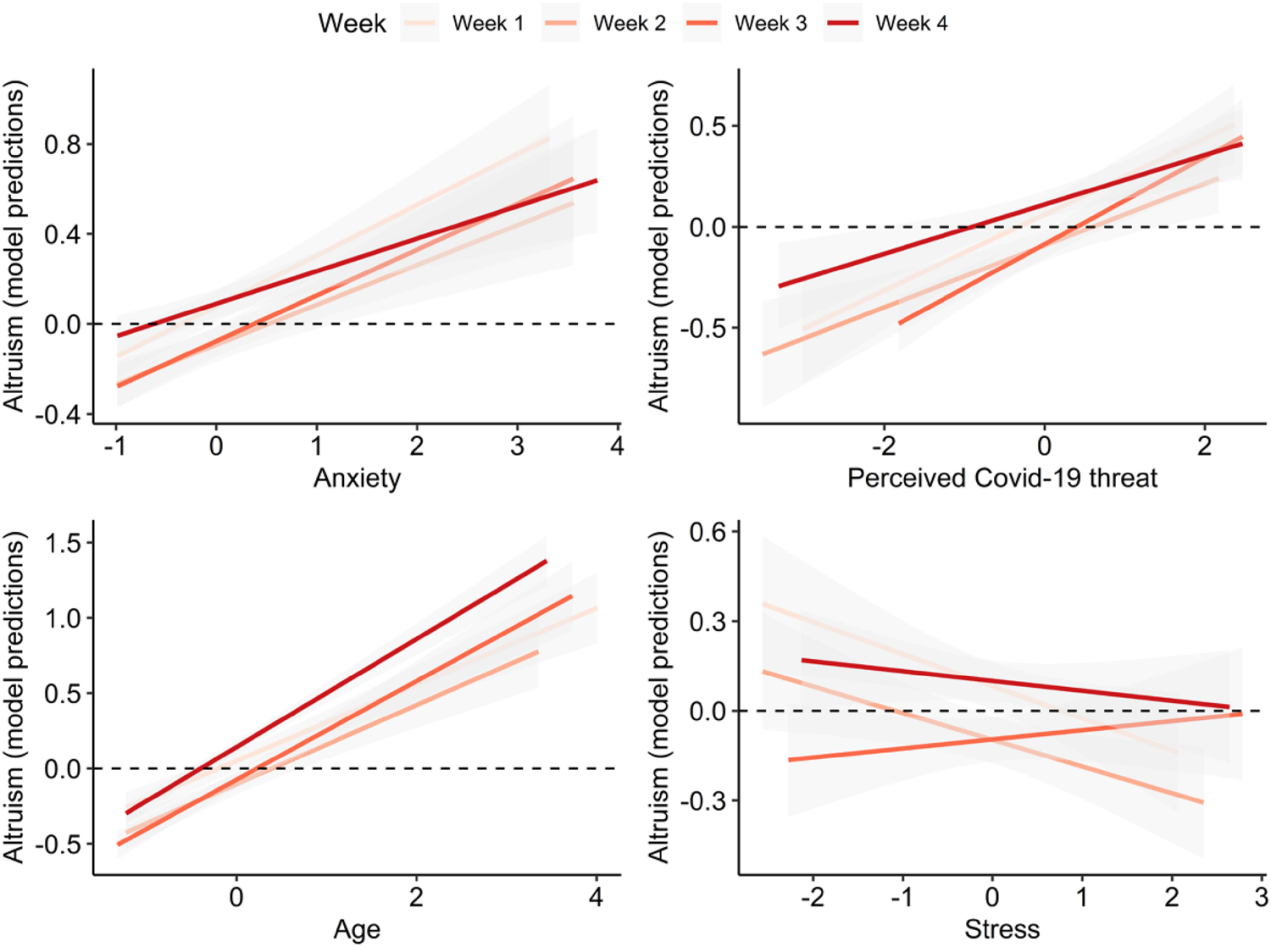
Model 2 predictions of everyday altruism (SRA) as a function of Anxiety (top left), Perceived COVID-19 threat (top right), Age (bottom left) and Stress (bottom right), depicting estimated random slopes per week.

We next examined the association between other demographic variables and everyday altruism, focusing on age, gender, socio-economic status and employment. Rather than introducing all these additional fixed effects in the main model, we instead first modelled them separately, with the goal of identifying those significantly associated with altruism. This strategy helped limit the complexity of the main model and avoid convergence problems (see Supplementary material for results of the model with all predictors, fixed effects of interest plus additional demographics, which did not converge but showed results in the same direction and significance as those reported here). Age was found to be positively associated with altruism, and employment was marginally negatively associated (Supplementary material). We thus added these variables to the main model (Model 2). Results showed that age, perceived COVID-10 threat, and anxiety were uniquely associated with increased everyday altruism (Table 2, Figs. 2B and 3). When using a simpler model with only a random intercept by week, in addition to these effects, we found depression, stress and unemployment were negatively associated with altruism (Supplementary material Table m3.3).

**Table 2:** Model 2 estimates (DV: Everyday altruism, SRA).

	Estimate	Std. Error	df	t value	Pr( >|t|)	CI_lower	CI_upper
(Intercept)	0.119	0.070	3.580	1.694	0.174	− 0.019	0.256
Perceived COVID-19 threat	0.156	0.051	4.674	3.090	0.030*	0.057	0.256
Stress	− 0.084	0.063	5.228	− 1.327	0.239	− 0.208	0.040
Anxiety	0.296	0.052	9.196	5.641	0.000*	0.193	0.399
Depression	− 0.132	0.069	5.115	− 1.915	0.112	− 0.268	0.003
Age	0.314	0.042	577.963	7.498	0.000*	0.232	0.396
Employment* = Student	− 0.120	0.119	8.789	− 1.009	0.340	− 0.352	0.113
Employment = Unemployed	− 0.416	0.149	3.231	− 2.786	0.063	− 0.708	− 0.123

*Reference class = Employed.

Overall, results showed higher perceived COVID-19 threat, and higher acute anxiety were robustly associated with increased everyday altruism, with some evidence emerging for a negative association with stress. This pattern of results can be interpreted as higher threat perception and acute defence during a crisis being linked with increased engagement in altruism, or alternatively that those higher in altruistic tendencies are more likely to experience threat and acute anxiety in response to a crisis.

### Modelling other indicators of altruism during the pandemic

We next examined whether the observed associations between everyday altruism, perceived COVID-19 threat, and anxiety would hold when using an indicator that specified altruistic action during the pandemic, namely the reported frequency of altruistic behaviours towards strangers or charity in the last few weeks. Results showed that anxiety and age were positively associated with reporting altruistic behaviours towards a stranger or charity during the COVID-19 pandemic (Table 3).

**Table 3:** Model 3 estimates (DV: Help stranger).

	Estimate	Std. error	df	t value	Pr( >|t|)	CI_lower	CI_upper
(Intercept)	0.040	0.081	3.994	0.493	0.648	− 0.120	0.200
Perceived COVID-19 threat	0.047	0.068	2.601	0.686	0.549	− 0.087	0.180
Stress	− 0.050	0.132	1.170	− 0.380	0.761	− 0.309	0.209
Anxiety	0.267	0.079	3.389	3.380	0.036*	0.112	0.422
Depression	− 0.137	0.155	1.068	− 0.883	0.532	− 0.441	0.167
Age	0.230	0.067	291.869	3.419	0.001*	0.098	0.362
Employment (1 = Student)^1^	0.099	0.162	16.485	0.611	0.549	− 0.219	0.417
Employmen (2 = Unemployed)	− 0.130	0.171	1.585	− 0.759	0.544	− 0.465	0.205

^1^Reference class = Employed.

We also replaced the SRA total score with that referring to donations only in the full model (Model 4). Results showed that anxiety and age remained significantly associated with increased donation behaviours. Additionally, Depression was negatively associated with those behaviours (Table 4).

**Table 4:** Model 4 estimates (DV: Donations).

	Estimate	Std. error	df	t value	Pr( >|t|)	CI_lower	CI_upper
(Intercept)	0.109	0.072	3.366	1.520	0.216	− 0.032	0.250
Perceived COVID-19 threat	0.129	0.051	4.868	2.534	0.054	0.029	0.228
Anxiety	0.210	0.061	5.125	3.471	0.017*	0.092	0.329
Depression	− 0.158	0.063	20.345	− 2.510	0.021*	− 0.281	− 0.035
Stress	− 0.027	0.067	5.632	− 0.398	0.705	− 0.157	0.104
Age	0.282	0.055	3.992	5.115	0.007*	0.174	0.391
Employment (1 = Student)^1^	− 0.039	0.125	6.831	− 0.313	0.764	− 0.285	0.207
Employment (2 = Unemployed)	− 0.443	0.149	3.336	− 2.975	0.051	− 0.735	− 0.151

^1^Reference class = Employed.

Finally, we re-ran the full model using the Prosocial Behavioral Intentions (PBI) score as dependent measure. We found no evidence of a significant association between prosocial intentions and any of our predictors (Supplementary material). Of note, this measure assesses the willingness to engage in future prosocial behaviour rather than self-reported performed behaviours. Also, visual inspection of the PBI data revealed skewed distributions, with the majority of scores concentrated in the upper end of the scale. This suggests participants generally rated their behavioural intentions as very highly prosocial (maybe due to social desirability), and variability in responses was low (see Supplementary material). This limitation of the measure could have contributed for the disparity in findings between prosocial intentions and the other altruistic measures.

### Associations among measures of prosociality and defensive emotions

**Figure 4:**
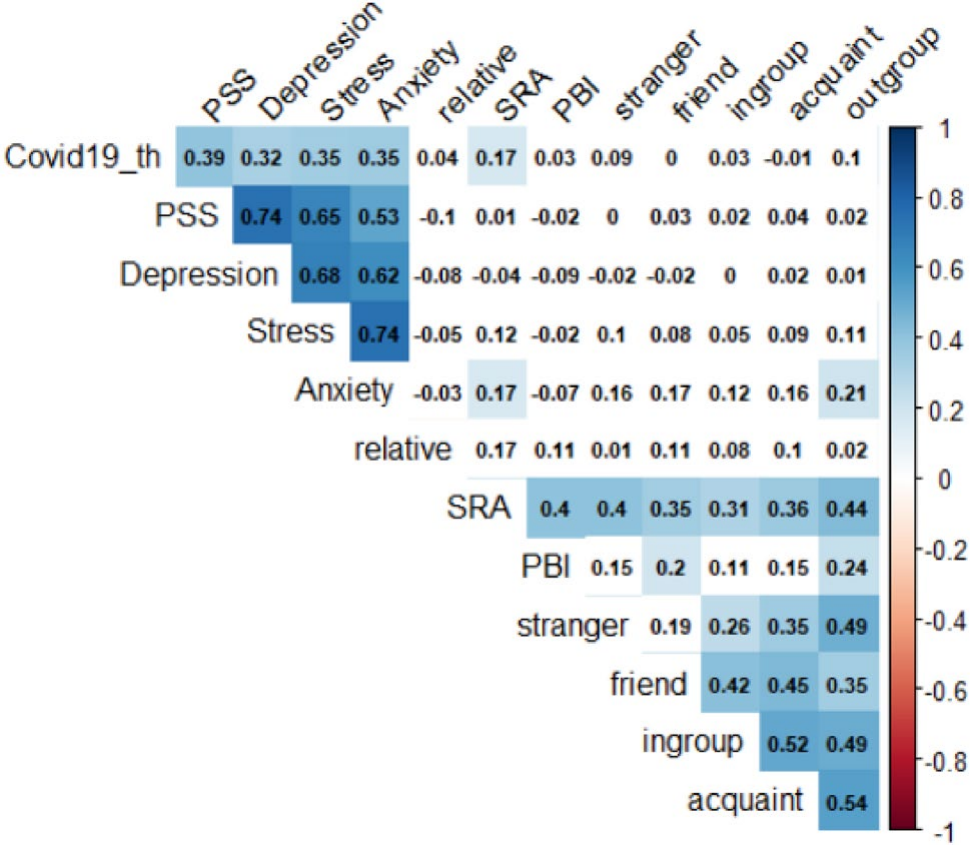
Zero-order correlations between indices of prosocial behaviour and defensive responses. The numbers in each cell correspond to the Pearson r coefficients. Cells with a white background correspond to correlations that did not survive correction for multiple comparisons. Covid19_th: Perceived COVID-19 threat; PSS: Perceived Stress Scale; Depression: DASS-21 Depression scale; Stress: DASS-21 Stress scale; Anxiety: DASS-21 Anxiety scale; SRA: Self-report Altruism; PBI: Prosocial Behavioural Intentions scale; stranger: Frequency of altruistic acts towards stranger; friend: Frequency of altruistic acts towards a friend; relative: Frequency of altruistic acts towards a relative; ingroup: Frequency of altruistic acts towards an ingroup member; outgroup: Frequency of altruistic acts towards an outgroup member; acquaint: Frequency of altruistic acts towards an acquaintance.

At an exploratory level, we examined the associations between everyday altruism, other measures of prosocial behaviour, and measures assessing defensive emotional states (anxiety and stress). Correlation analyses (corrected for multiple comparisons, all reported *p*s < 0.00065) revealed moderate positive correlations between everyday altruism and other indicators of prosocial behaviour, including prosocial behavioural intentions, and frequency of altruistic behaviours towards friends, acquaintances, strangers, ingroup and outgroup members (Fig. 4). Perceived COVID-19 threat was positively associated with state measures of anxiety and stress. In line with the main analysis, everyday altruism was positively correlated with perceived COVID-19 threat and anxiety. There results point to a coherent network of associations between measures assessing defensive and prosocial behaviour, respectively, and further support a unique link between altruistic behaviour and acute defensive responses to the pandemic.

## Discussion

This study investigated the association between everyday altruistic behaviour, changing level of COVID-19 threat over time (objective threat imminence), and type of defensive emotions experienced by individuals during this period (perceived threat imminence). It has been proposed that emotional states like stress, anxiety, fear and panic can be understood as reflecting contiguous defensive responses to varying levels of perceived threat^17,19^. Based on observations that defensive responses to increased threat imminence^23^ and acute stress^3^ may facilitate prosocial action in experimental settings, we hypothesized that experiencing more acute defensive emotions during the pandemic would be associated with higher levels of self-reported altruism. Our results supported this hypothesis, showing that individuals reporting higher perceived COVID-19 threat and experiencing higher acute anxiety (conceptualized as high physiological arousal and feelings of being close to panic), also reported higher everyday altruism. These associations persisted while controlling for additional variables such as age, and employment status. The association between everyday altruism and acute anxiety was particularly strong and consistent both across, and within each week (when each week was analysed separately, acute anxiety emerged as a robust predictor of everyday altruism—see Supplementary material), suggesting that acute defensive states may be particularly predictive of altruistic outcomes.

Our findings are consistent with previous reports that acute social stressors promote prosocial behaviour. For instance, individuals submitted to the Trier Social Stress Test, a paradigm that increases salivary cortisol and heart rate in experimental studies, have been shown to subsequently display increased levels of trustworthiness and sharing^9,30^, empathy for others’ pain^11^, and self-reported emotional empathy^31^. Of note, despite the different terminology, “acute stress” in these experimental studies has a physiological manifestation and is more closely related to our operationalization of acute anxiety. That acute social stressors promote affiliative behaviour has often been interpreted with reference to the “tend-and-befriend” hypothesis by Taylor^32,33^. This hypothesis proposes an oxytocin-mediated biobehavioural system that, on the one hand, fosters affiliative behaviours that maximize species survival (tending), and, on the other, reduces stress through the establishing of social bonds (befriending). The “tend-and-befriend” proposal thus highlights the role of oxytocin in promoting social bonds and, as a result, in dampening defensive responses in social stressful situations. However, animal and human research have demonstrated that oxytocin plays a more general role in regulating individual defensive behaviour in non-social contexts as well^34^. For instance, oxytocinergic neurons in the central amygdala are implicated in allowing transitions between freezing and active defense (fight-or-flight) in response to imminent danger^35,36^. Moreover, in rodents, the oxytocin-mediated ability to inhibit freezing in favor of active threat coping behaviours is necessary to allow females to engage in offspring defense^37^. In light of these data, one possibility is that acute defensive states enable prosocial action regardless of the social nature of the stressor, by recruiting active coping mechanisms also implicated in dealing with first-hand threats. This is consistent with recent experimental data showing that the induction of fight-or-flight states by increasing threat imminence promotes costly helping decisions in humans^23^. It is also consistent with previous theoretical accounts suggesting acute physiological stress (versus diffuse or chronic stress) promotes caregiving specifically in situations of immediate need^8,38^. While open questions remain regarding the underlying mechanisms, our results demonstrate a positive relation between acute defensive states and prosocial behaviour, which generalizes beyond time bounded laboratory experiments to real-life threatening contexts that extend over time.

Interestingly, we found either a non-significant or negative association (depending on how the models were defined) between altruism and stress, characterized here by diffuse anxiety and feelings of uncontrollability and unpredictability. One possible interpretation for this finding, is that the association between altruism and threat perception is modulated by perceived threat imminence. i.e., altruism is differently associated with defensive emotions typical of higher perceived threat imminence (acute anxiety), and those eventually elicited when threat imminence is perceived to be lower (stress). It should be noted however, that our questionnaire measures do not directly assess perceived threat imminence. Rather, our measures of stress mainly tapped onto feelings of controllability and unpredictability. Therefore, our interpretation, and the potentially dissociable association between altruism and perceived threat imminence, warrants further examination in more controlled experiments (e.g.,^23^). Additionally, because the (negative) relation between stress and altruism was not as robust as the (positive) relation with acute anxiety, replication of the stress effect in future well-powered studies is especially warranted.

One aspect to consider is that our main indicator of altruism, the Self-report Altruism Scale (SRA), does not specify a time window for the occurrence of behaviour, and thus altruistic behaviours reported by participants may have included actions not necessarily undertaken during the pandemic. We have accounted for this in the analysis by testing our model with an index of altruistic behaviours performed during the pandemic, having confirmed the same pattern of results with acute anxiety. In addition, the SRA includes actions that could be difficult to perform during the lockdown. Our analysis also accounted for this aspect, demonstrating results in the same direction with acute anxiety while using only SRA items related to donation behaviours, which although still hindered to some extent by the lockdown context (e.g., donating blood)could more easily be undertaken (e.g., charity donations). Despite these limitations (which we have accounted for in the analysis), the SRA has the advantage of being the only validated and well-established measure of self-reported altruistic behaviour in our study. Crucially, even if understood as trait measure, our findings with the SRA suggest that altruistic dispositions are associated with the propensity to experience acute anxiety (but not stress) during a real-life crisis. Such trait-level association has important theoretical implications, since it further supports a link between neurobiological systems responsible for defensive responding and those implicated in empathy and care. Future research would benefit from considering additional trait level moderators of interest in the association between defense and altruism, such as individual differences in threat sensitivity and dispositional empathy.

We had also hypothesized that increased COVID-19 threat over time (i.e., increasing number of cases and fatalities) would result in increasing indices of everyday altruism at the population level. Our results for this hypothesis were inconclusive. Although donations decreased from Week 1 to Week 2, no clear indications of increase or decrease in altruism emerged over the 4 weeks of data collection. Critically, our inability to detect meaningful alterations in altruism may have been due to the fact that, despite the increasing number of cases and fatalities, subjective perception of COVID-19 threat did not significantly change over those 4 weeks. There are several potential, not mutually exclusive reasons for the disparity between objective and subjectively perceived threat. For one, the use of different samples each week may have compromised our power to capture changes over time. Another likely reason is that our study had insufficient temporal resolution to detect changes in subjective threat perception that accompanied the course of the pandemic. It is possible that individuals perceived the COVID-19 situation to be substantially more severe prior to the first data collection on March 24th, and that perception became relatively stable over the following weeks. Indeed, in the early stages of the pandemic in the United States, information about the coronavirus and its transmission was still limited and unclear. The perception of danger in these early stages was therefore presumably higher, as suggested by the outages of toilet paper and other goods at supermarkets (https://www.mckinsey.com/industries/consumer-packaged-goods/our-insights/us-food-supply-chain-disruptions-and-implications-from-covid-19), and increase in gun sales across the country (https://www.theguardian.com/us-news/2021/may/31/us-gun-sales-rise-pandemic). Some research supports this interpretation, with data suggesting that more substantial changes in COVID-19 risk perception may have occurred prior to the beginning of our study^39,40^. Another possibility is that objective indices of COVID-19 threat, like number of cases and deaths, are not the only contributing factor for subjective threat perception. For instance, knowledge about the measures that are being implemented to control the virus spread may modulate how severe individuals perceive the situation to be. In that regard, the start of the lockdown in the US during the data collection period may have mitigated the perceived threat. Accounting for the state of residence of the participants could also have improved the sensitivity of the analysis to detect changes in threat perception, since the evolution of the pandemic was quite heterogeneous across different locations. Our dataset had an uneven number of cases per US state in each week, substantially limiting statistical power of any spatially-based analyses. However, even accounting for the number of COVID-19 cases specifically in the participants’ state of resident, we did not find indication that it was associated with perceived COVID-19 threat (*r* = 0.006, *p* = 0.88). In sum, given that we cannot discount the influence of between-sample variation in our data, that our time resolution may have been insufficient, and that perceived threat did not change significantly over the 4 weeks, our study was inconclusive to assess a potential link between changing threat imminence over time and population levels of altruistic behaviour. Future research on population-level effects would benefit from including information regarding location, as well as additional demographic aspects, such as having children or not, and health status of the participants.

As a final note, it is worth pointing out the strong association that emerged between age and altruism. This association has been previously documented, e.g.,^41–46^, including in relation to situations of acute stress^47^. The positive association between age and altruism is likely explained by a combination of biological and sociocultural factors, which are out of the scope of the present study. Of note, we did not find indication that this association was driven by other demographical aspects (such as employment or socio-economic level), which likely covary with age but were controlled for in our analysis. The inclusion of additional variables, such as having children and health status, could help to further clarify the apparent positive impact of age on everyday altruism. It should also be noted that the strength of the age effect we found could, to some extent, be driven by how the SRA scale is formulated, i.e., in measuring accumulated behaviour over time, older participants might by default report more engagement in altruistic behaviours.

Some specificities of our study should be acknowledged. At the conceptual level, it is important to note that, while our operationalization of different defensive states was informed by the threat imminence framework, it does not entirely match how threat imminence has been previously manipulated in experimental studies. Specifically, experimental studies have typically manipulated features of threatening stimuli (e.g., spatial proximity), thereby changing actual threat imminence. Conversely, in our study, we took two approaches: first, we inferred threat imminence indirectly based on the number of COVID-19 cases; and second, we quantified individual defensive responses that are thought to reflect variation in perceived threat imminence. This difference in *actual* versus *perceived* threat should be taken into account when interpreting our results, although it is noteworthy that our results were consistent with a recent experimental study that directly manipulated threat imminence^23^.

A related limitation refers to the self-report measures used to index different defensive states. While these measures (DASS-21 scales and Perceived Stress Scale) tapped into dimensions closely related to varying threat imminence (e.g., predictability/controllability, versus high arousal and feelings of panic), they did not provide a direct measure of perceived threat imminence. Moreover, our ability to capture extreme levels of stress and anxiety may have been limited by the fact that high scorers in those measures (e.g., individuals with small children at home, or caring for ill relatives) may be less likely to participate in an online study. Finally, it is worth acknowledging the difficulty in measuring altruistic behaviour during the pandemic due to, among other reasons, reliance on self-report, and behavioural restrictions imposed by lockdown.

## Methods

### Participants

602 participants were recruited through the Prolific participant pool (Prolific.co). The sample size was decided based on resource availability (largest N possible with available resources), and not a formal power calculation. To mitigate the lack of a power calculation, we performed simulation-based power analysis after completing the study, fixing the sample size to the one we used to determine our power to detect effect sizes of different magnitudes. This procedure provides an estimate of how much power our study had, without relying on the obtained effects sizes^48,49^. This analysis suggested that our sample size and design would have between 88 and 94% power to detect a 0.3 effect size (d) (details on this analysis and power curve in Supplementary material).

Participant inclusion criteria were Nationality and residence in the United States, and fluency in English. Data were collected at 4 timepoints, over a 4-week period: 24-03-2020 (Week 1, n = 150), 31-03-2020 (Week 2, n = 150), 07-04-2020 (Week 3, n = 150), and 14-04-2020 (Week 4, n = 151). Data collection took place at approximately 20:30 (CEST) each week (i.e., the study was set to active on Prolific at that time). The collection of 4 samples at different timepoints (rather than N = 600 at one timepoint) provided a way of examining the robustness of the findings across independent samples, which combined with the statistical approach adopted (Generalized Linear Mixed Effects Models, see below) has been suggested to improve the generalizability of research findings^50^.

**Figure 5:**
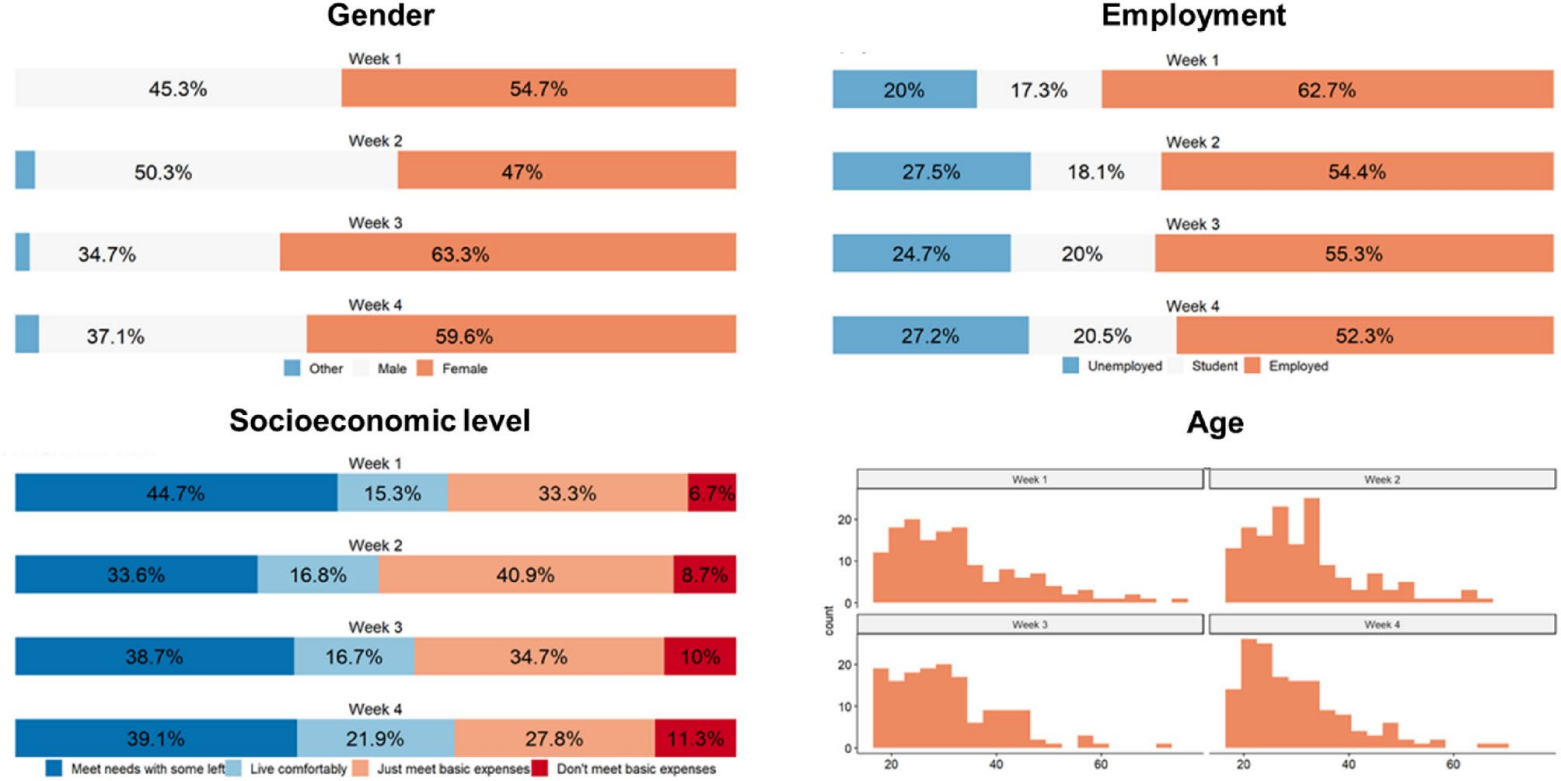
Demographic information for the sample collected in each week.

Participants completed an online survey consisting of several questionnaire measures, in addition to demographical information, namely age, gender, employment status, state of residence, socio-economic status and education level. The full survey (available here: https://osf.io/c8zhn/) took about 15 min to complete. One entry was excluded because the data appeared to be a duplicate (i.e., two entries had identical responses in open-ended fields for participant comments and questions, and all demographics except age were identical). The data seemed otherwise valid, and thus only the first entry was retained. Another participant was excluded due to incomplete information regarding state of residence, yielding a total of 600 participants in the final sample (*M*_*age*_ = 31.16, *SD*_*age*_ = 10.70, 56.1% female, 2.0% other; Fig. 5). All participants provided informed consent prior to participating.

All methods and experimental protocols were carried out in accordance with relevant guidelines and regulations, and approved by the Karolinska Institutet (Sweden) and the Etikprövningsmyndigheten (Ethics review authority).

### Questionnaire measures

#### Everyday altruism

Everyday altruism was measured through the widely used Self-Report Altruism Scale^51^ (*M* = 50.83, SD = 11.44, range = [24, 98]; SRA normative data from^51^ mean = 55.40 and SD = 10.57), in which participants rate the frequency (1 = never, 5 = very often) with which they engaged in 20 different everyday altruistic behaviours (e.g., “I have given money to a charity”, “I have offered my seat on a bus or train to a stranger who was standing”). These ratings are believed to reflect both the frequency of performed altruistic acts, and the individual’s endorsement of helping others in everyday situations^51^.

The SRA does not specify a timeframe for the occurrence of altruistic actions, thereby including both acts performed in the weeks leading up to the moment of data collection (i.e., during the pandemic), as well as prior in the participant’s life. To obtain more detailed information about self-reported altruistic actions specifically during the timeframe of the study, we created 5 additional items in which participants rated the frequency of altruistic actions towards different targets: “In the last few weeks, think of anything you may have done to help or benefit another person, without any benefit for yourself. Examples would be donating money, clothes or food, caring for someone who was unwell, assisting someone in a task they were unable to complete on their own, etc. If any of these or similar behaviours occurred in the last few weeks, how many of those behaviours were directed at…”. Subsequently, participants rated the amount of such behaviours that were directed at a relative (*Help relative*), a friend (*Help friend*), an acquaintance (*Help acquaintance*), a stranger or a charity (*Help stranger*), a person having a lot in common with (*Help ingroup*), and a person having nothing or very little in common with (*Help outgroup*; 1 = none, 4 = all). The idea to include these measures arose after the study had been initiated, and therefore these additional items are only available for Weeks 3 and 4 (n = 300).

Additionally, to assess prosocial intentions (rather than performed behaviour), we used the Prosocial Behavioural Intentions Scale^52^, wherein individuals rate how likely they would be to engage in 4 types of prosocial actions (“Comfort someone I know after they experience a hardship”, “Help a stranger find something they lost, like their key or a pet”, “Help care for a sick friend or relative”, “Assist a stranger with a small task (e.g., help carry groceries, watch their things while they use the restroom”).

#### Perceived COVID-19 threat and defensive emotional responses

Perceived COVID-19 threat was operationalized through the COVID-19 Risk Perception Scale (RP) developed by Wise and collaborators^26^ (M = 45.27, range = [10, 70]), which measures self-reported feelings and thoughts about the COVID-19 pandemic, including perceived risk of infection and economic repercussions (e.g., “How likely do you think you are to catch the virus”, 1 = min, 7 = max).

Defensive emotional responses were assessed through the Perceived Stress Scale-10 (PSS-10^27^) and the Depression Anxiety Stress Scales (DASS-21^28^). The PSS-10 (M = 20.73, range = 3, 40]) includes 10 items assessing experienced stress, specifically the degree to which individuals perceived their lives to be unpredictable, uncontrollable, and overloaded in the past month (e.g., “How often have you been upset because something that happened unexpectedly?”, 1 = never, 5 = very often).

The DASS-21 includes 21 items assessing states of stress, anxiety and depression over the past week, using a 1 (Did not apply to me at all), to 4 (applied to me very much or most of the time) scale. The Stress scale (M = 15.29, range = [0, 42]) measures difficulty relaxing, unspecific nervousness, and being easily upset and impatient (e.g., “I found it hard to wind down). The Anxiety scale (M = 8.23, range = [0, 40]), on the other hand, assesses states of acute anxiety, characterized by high autonomic arousal and skeletal muscle effects (e.g., “I was aware of dryness of my mouth”, “I felt close to panic”), presumably more characteristic of higher perceived threat imminence (post-encounter and circa-strike contexts). Finally, the Depression scale (M = 14.2, range = [0, 42]) assessed feeling of dysphoria, hopelessness, devaluation of life, self-deprecation, lack of interest, anhedonia, and inertia (e.g., “I couldn’t seem to experience any positive feeling at all”). Although not the focus of the present study, the depression scale was included to account for shared variance with stress and anxiety.

All questionnaires were used at all time points, with the exception of the items assessing frequency of altruistic behaviours towards different targets, which were only measured on Weeks 3 and 4.

The COVID-19 Data Repository by the Center for Systems Science and Engineering (CSSE) at Johns Hopkins University (https://github.com/CSSEGISandData/COVID-19/) was used as a resource for obtaining objective COVID-19 data during the time window of the study, including the number of cases and fatalities per state in the United States. Objective COVID-19 threat was operationalized by the number of total confirmed cases per state of residence.

### Statistical analysis

#### Changes in everyday altruism and perceived COVID-19 threat over time

Our first question was whether increased COVID-19 threat over time was accompanied by higher frequency of self-reported altruistic behaviours at the population level. To test our hypothesis, we performed one-way ANOVAs, using perceived COVID-19 threat and everyday altruism as dependent variables. Our main indicator of everyday altruism was the SRA score. Because some of the items of the SRA describe actions that may be difficult to perform during a lockdown (e.g., helping a stranger with care troubles), we repeated the ANOVA using as dependent variable a subset of SRA items referring to donations, given that the ability to carry out those behaviours should be less affected (items: 4. I have given money to a charity, 5. I have given money to a stranger who needed it (or asked me for it), 6. I have donated goods or clothes to a charity, 7. I have done volunteer work for a charity, 8. I have donated blood; Cronbach’s alpha for SRA donations = 0.7). In each ANOVA, we assessed whether Perceived COVID-19 threat and indices of everyday altruism (SRA and SRA-donations) varied over the 4 weeks of data collection.

#### Modelling everyday altruism (SRA) as a function of perceived COVID-19 threat and defensive emotions

Our second question was whether experiencing different defensive emotional states during the pandemic was associated with self-reported altruism at the individual level. To assess this question, we adopted a Generalized Linear Mixed Models (GLMMs) approach. GLMMs explicitly model variance in a hierarchical fashion, in keeping with the hierarchical structure of datasets in which different observations belong to different groups or were collected at different timepoints (e.g., week). Thus, this approach allowed us to account for variation in everyday altruism (SRA; dependent variable) that was explained by our variables of interest (fixed effects), as well as by random sampling of timepoint (random effect). Initially, both state of residence and week were included as random effects. However, given (a) the limited and unbalanced number of observations from some US states, (b) the high number of random effect estimates necessary to model state as a random effect, and c) the negligible variance explained by the random effect of state, we opted to exclude state from the model. Week was modelled as a random effect, with random intercept and slope per each fixed effect (details in Supplementary material).

Key fixed effects of interest included in Model 1 were: (1) *perceived COVID-19 threat* (COVID-19 RP scale); (2) *stress* (PSS), defined as feelings of uncontrollability and unpredictability, which are more characteristic in response to situations of lower threat imminence; and, (3) *anxiety* (DASS-21 Anxiety), defined as a state of high autonomic arousal, acute anxiety and panic, consistent with emotional responses to threats perceived as predictable and imminent. Of note, the PSS score was chosen over the DASS-21 Stress scale to index stress due to the stronger correlation between DASS-21 Stress and Anxiety (Fig. 4). Importantly, we verified that including DASS-21 Stress instead of PSS in the models did not change the direction of results (see Supplementary material). Although not the focus of our hypotheses, Depression was also included as a fixed effect, since it is thought to have important associations with stress and anxiety-related responses^29^. Including Depression in our models was critical to account for shared variance with stress and anxiety, especially given we hypothesized the opposite association between altruism and acute defensive emotions (positive) to that previously described for Depression (negative)^53,54^. Multicollinearity diagnostics using the Kappa statistic and Variance Inflation Factor (VIF) did not suggest problematic collinearity when stress, anxiety and depression were included in the same models (all kappas < 3.66; all VIFs < 5). Also, including Depression did not affect the direction or significance of the main findings (Supplementary material).

In addition to the fixed effects of interest, we modelled demographic variables with a presumed association with altruistic motivation, namely age, gender, socio-economic status and employment (Model 2). Briefly, (1) *age* has been shown before to be positively associated with prosocial tendencies^55,56^; (2) *gender* may modulate prosocial behaviour^6,57^, particularly in stressful situations^32^; and (3) *socio-economic* and *employment* status could compromise one’s ability to carry out some types of altruistic acts (e.g., money donations), and affect subjective wellbeing, which has been previously linked with altruism^7^.

### Modelling other indicators of altruism during the pandemic

Because our main measure of everyday altruism (SRA) does not specify a timeframe for the reported behaviours, we performed additional GLMM using the *Help stranger* item as dependent variable (Model 3). This item assessed the frequency of altruistic acts towards a stranger or charity in the last few weeks. This measure was chosen because: (1) it specifies a timeframe consistent with the pandemic; (2) in assessing altruism towards a stranger/charity, it was deemed a “purer” measure of altruism than the items assessing frequency of altruistic actions towards relatives, friends or acquaintances, which can be inherently rewarding, or reciprocity-based.

Another limitation of using the SRA is that some of its items describe actions that may be difficult to perform during a lockdown (e.g., helping a stranger with care troubles). To minimize this potential concern, we also repeated the GLMM analysis using the SRA-donations as dependent variable (Model 4).

Finally, we repeated the analysis using the Prosocial Behavioral Intentions scale (PBI).

The description of the different models tested is presented together with the respective results in the next section, to improve readability. Also, for the sake of conciseness, not all steps of the modelling were included in the main text. All modelling steps and results, code, model assumptions, as well as an analysis assessing the robustness of the effects within each week are available in Supplementary material.

#### Correlations among measures of prosociality and defensive emotions

Finally, at an exploratory level, we used zero-order Pearson correlations to examine associations between self-reported altruism, other measures of prosocial behaviour (Prosocial Behavioral Intentions Scale, items assessing altruistic behaviours towards different targets), and measures assessing defensive emotional states (anxiety and stress). These analyses were performed across all weeks (N = 600), except for the items assessing altruism towards different targets, for which only two Week 2 and 3 were available (N = 300). Correlation analyses between these items were Sidak-corrected for multiple comparisons.

All analyses were implemented in R, and the GLMMs performed using lme4 package^58^ with standardized variables. The significance level was set at α < 0.05. In line with open science practices, all analytical decisions and steps are detailed in Supplementary material, and all data, study materials and code are publicly available on the OSF project page (https://osf.io/c8zhn/).

## Supplementary Information

Supplementary Information.

## Data Availability

All data, code and study materials are publicly available on OSF (https://osf.io/c8zhn/).

## References

[1] Atrooz F, Liu H, Salim S (2019). Stress, psychiatric disorders, molecular targets, and more. Prog. Mol. Biol. Transl. Sci..

[2] Starcke K, Brand M (2012). Decision making under stress: A selective review. Neurosci. Biobehav. Rev..

[3] Buchanan, T. W. & Preston, S. D. Stress leads to prosocial action in immediate need situations. *Front. Behav. Neurosci.* **8**, (2014). 10.3389/fnbeh.2014.00005 PMC3897879 24478652

[4] Feinberg, J. Psychological egoism. in *Ethical Theory: An Anthology* (Wiley, 2014).

[5] Mattis JS (2009). The social production of altruism: Motivations for caring action in a low-income urban community. Am. J. Community Psychol..

[6] Sisco MR, Weber EU (2019). Examining charitable giving in real-world online donations. Nat. Commun..

[7] Brethel-Haurwitz KM, Marsh AA (2014). Geographical differences in subjective well-being predict extraordinary altruism. Psychol. Sci..

[8] Preston SD (2013). The origins of altruism in offspring care. Psychol. Bull..

[9] von Dawans B, Ditzen B, Trueg A, Fischbacher U, Heinrichs M (2019). Effects of acute stress on social behavior in women. Psychoneuroendocrinology.

[10] Singer N (2017). Acute psychosocial stress and everyday moral decision-making in young healthy men: The impact of cortisol. Horm. Behav..

[11] Tomova L (2017). Increased neural responses to empathy for pain might explain how acute stress increases prosociality. Soc. Cogn. Affect. Neurosci..

[12] Buchanan TW, Bagley SL, Stansfield RB, Preston SD (2012). The empathic, physiological resonance of stress. Soc. Neurosci..

[13] Starcke K, Polzer C, Wolf OT, Brand M (2011). Does stress alter everyday moral decision-making?. Psychoneuroendocrinology.

[14] Zhang Q, Ma J, Nater UM (2019). How cortisol reactivity influences prosocial decision-making: The moderating role of sex and empathic concern. Front. Hum. Neurosci..

[15] Fanselow, M. S. & Lester, L. A functional behavioristic approach to aversively motivated behavior: predatory imminence as a determinant of the topography of defensive behavior. in *Evolution and Learning, Evol.* 185–212 (1988).

[16] Blanchard, R. J. & Blanchard, D. C. Anti-predator defense as models of animal fear and anxiety. in *Fear and Defence* 89–108 (Harwood Academic Publishers, 1990).

[17] Mobbs D, Headley DB, Ding W, Dayan P (2020). Space, time, and fear: survival computations along defensive circuits. Trends Cogn. Sci..

[18] Hamm AO (2020). Fear, anxiety, and their disorders from the perspective of psychophysiology. Psychophysiology.

[19] McNaughton N, Corr PJ (2004). A two-dimensional neuropsychology of defense: Fear/anxiety and defensive distance. Neurosci. Biobehav. Rev..

[20] Ehlers MR, Nold J, Kuhn M, Klingelhöfer-Jens M, Lonsdorf TB (2020). Revisiting potential associations between brain morphology, fear acquisition and extinction through new data and a literature review. Sci. Rep..

[21] Perkins AM, Cooper A, Abdelall M, Smillie LD, Corr PJ (2010). Personality and defensive reactions: Fear, trait anxiety, and threat magnification. J. Pers..

[22] Perkins AM, Corr PJ (2006). Reactions to threat and personality: Psychometric differentiation of intensity and direction dimensions of human defensive behaviour. Behav. Brain Res..

[23] Vieira JB, Schellhaas S, Enström E, Olsson A (2020). Help or flight? Increased threat imminence promotes defensive helping in humans. Proc. Biol. Sci..

[24] Vieira JB, Olsson A (2022). Neural defensive circuits underlie helping under threat in humans. eLife.

[25] Philippe Rushton J, Chrisjohn RD, Cynthia Fekken G (1981). The altruistic personality and the self-report altruism scale. Personal. Individ. Differ..

[26] Wise T, Zbozinek TD, Michelini G, Hagan CC, Mobbs D (2020). Changes in risk perception and self-reported protective behaviour during the first week of the COVID-19 pandemic in the United States. R. Soc. Open Sci..

[27] Cohen S, Kamarck T, Mermelstein R (1983). A global measure of perceived stress. J. Health Soc. Behav..

[28] Lovibond S, Lovibond P (1995). Manual for the Depression Anxiety Stress Scales.

[29] Kalin NH (2020). The critical relationship between anxiety and depression. Am. J. Psychiatry.

[30] von Dawans B, Fischbacher U, Kirschbaum C, Fehr E, Heinrichs M (2012). The social dimension of stress reactivity: Acute stress increases prosocial behavior in humans. Psychol. Sci..

[31] Wolf OT (2015). Enhanced emotional empathy after psychosocial stress in young healthy men. Stress.

[32] Taylor SE (2000). Biobehavioral responses to stress in females: Tend-and-befriend, not fight-or-flight. Psychol. Rev..

[33] Taylor, S. E. Tend and befriend: Biobehavioral bases of affiliation under stress. *Curr. Dir. Psychol. Sci.* (2016).

[34] Neumann ID, Landgraf R (2012). Balance of brain oxytocin and vasopressin: Implications for anxiety, depression, and social behaviors. Trends Neurosci..

[35] Terburg D (2018). The basolateral amygdala is essential for rapid escape: A human and rodent study. Cell.

[36] Tovote P (2016). Midbrain circuits for defensive behaviour. Nature.

[37] Rickenbacher E, Perry RE, Sullivan RM, Moita MA (2017). Freezing suppression by oxytocin in central amygdala allows alternate defensive behaviours and mother-pup interactions. Elife.

[38] Preston, S. D. The evolution and neurobiology of heroism. in *The Handbook of Heroism and Heroic Leadership* (Taylor & Francis/Routledge, 2016).

[39] Ayers, J. D. *et al. How is the COVID-19 pandemic affecting cooperation?* (2020). 10.31234/osf.io/pk6jy.

[40] Wise, T., Zbozinek, T. D., Michelini, G., Hagan, C. C. & mobbs, dean. *Changes in risk perception and protective behavior during the first week of the COVID-19 pandemic in the United States*. (2020). 10.31234/osf.io/dz428. 10.1098/rsos.200742 PMC7540790 33047037

[41] Beadle JN, de la Vega CE (2019). Impact of aging on empathy: Review of psychological and neural mechanisms. Front. Psychiatry.

[42] Cutler, J. *et al.* Ageing disrupts reinforcement learning whilst learning to help others is preserved. *bioRxiv* 2020.12.02.407718 (2020). 10.1101/2020.12.02.407718.

[43] Freund AM, Blanchard-Fields F (2014). Age-related differences in altruism across adulthood: Making personal financial gain versus contributing to the public good. Dev. Psychol..

[44] Lockwood, P. *et al.* Ageing increases prosocial motivation for effort. 10.31234/osf.io/8c5ra (2020). 10.1177/0956797620975781 PMC7611497 33860711

[45] Rosi A, Nola M, Lecce S, Cavallini E (2019). Prosocial behavior in aging: Which factors can explain age-related differences in social-economic decision making?. Int. Psychogeriatr..

[46] Cutler J, Nitschke JP, Lamm C, Lockwood PL (2021). Older adults across the globe exhibit increased prosocial behavior but also greater in-group preferences. Nat. Aging.

[47] Sparrow EP, Armstrong BA, Fiocco AJ, Spaniol J (2019). Acute stress and altruism in younger and older adults. Psychoneuroendocrinology.

[48] DeBruine, L. & Barr, D. J. *Understanding mixed effects models through data simulation*. (2019). 10.31234/osf.io/xp5cy.

[49] Green P, MacLeod CJ (2016). SIMR: An R package for power analysis of generalized linear mixed models by simulation. Methods Ecol. Evol..

[50] Yarkoni, T. The Generalizability Crisis. (2019). 10.31234/osf.io/jqw35. 10.1017/S0140525X20001685 PMC10681374 33342451

[51] Rushton, J. P., Chrisjohn, F. R. D. & Fekken, G. C. *The Altruist Ic Personal Ity and the Self-Report Altruism Scale**. (1981).

[52] Baumsteiger R, Siegel JT (2019). Measuring prosociality: The development of a prosocial behavioral intentions scale. J. Pers. Assess..

[53] Frick A, Thinnes I, Hofmann SG, Windmann S, Stangier U (2021). Reduced social connectedness and compassion toward close others in patients with chronic depression compared to a non-clinical sample. Front. Psychiatry.

[54] Kupferberg A, Bicks L, Hasler G (2016). Social functioning in major depressive disorder. Neurosci. Biobehav. Rev..

[55] Matsumoto Y, Yamagishi T, Li Y, Kiyonari T (2016). Prosocial behavior increases with age across five economic games. PLoS ONE.

[56] Sze JA, Gyurak A, Goodkind MS, Levenson RW (2012). Greater emotional empathy and prosocial behavior in late life. Emot. Wash. DC.

[57] Willer R, Wimer C, Owens LA (2015). What drives the gender gap in charitable giving? Lower empathy leads men to give less to poverty relief. Soc. Sci. Res..

[58] Bates D, Mächler M, Bolker B, Walker S (2015). Fitting linear mixed-effects models using lme4. J. Stat. Softw..

