# Supplementary material for: Acute anxiety during the COVID-19 pandemic was associated with higher levels of everyday altruism
Source: Sci Rep. 2022 Nov 3;12:18619. doi: 10.1038/s41598-022-23415-2 (PMC9632580; doi:10.1038/s41598-022-23415-2)
Supplement: Supplementary file 1 — Supplementary Information. [file 41598_2022_23415_MOESM1_ESM.pdf]

Supplementary Material
Perceived threat and acute anxiety during the Covid-19 pandemic were associated with increased levels of everyday altruism

Additional questionnaire measures

Additional measures were included in the survey, namely assessing: attitudes towards ingroup and outgroup, political ideology, and preferred sources of information about the COVID-19 pandemic. These measures are outside the scope of the present paper, and not reported here, but are available on the OSF project page (https://osf.io/c8zhn/).

Supplementary analyses

1. Model testing and selection Before testing our model, we first standardised all continuous predictors, and the continuous dependent variable. Our dependent variable was Self-Reported Altruism, and the 4 predictors (fixed effects) were: COVID19 Perceived threat (risk perception, RP, in the code), Perceived Stress (PSS), Anxiety, and Depression.

Model 1 - Perceived COVID-19 threat, stress, anxiety and depression The full model contained the 4 main predictors of interest (fixed effects), plus a random intercept and random slope for each predictor nested by Week. Initially, we wanted to model random intercepts and random slopes by State as well, but because we had limited data nested per state, we decided the model did not have enough information per State to estimate these parameters. For instance, we would require 230 random effects estimates if we included State as a random effect in the model. Additionally, the random effects estimates for State can hardly model any additional random variance at all. As an example, the random effects coefficients for the 46 intercepts predicted for each state were all zero.

```r
ranef(lmer(SRA ~ RP + PSS + Anxiety + Depression +  (1|State), data=CVA_full))
```

```
## $State
##    (Intercept)
## AK           0
## AL           0
## AR           0
## AZ           0
## CA           0
## CO           0
## CT           0
## DC           0
## DE           0
## FL           0
## GA           0
## HI           0
## IA           0
```

```
## ID           0
## IL           0
## IN           0
## KA           0
## KY           0
## LA           0
## MA           0
## MD           0
## ME           0
## MI           0
## MN           0
## MO           0
## MS           0
## NC           0
## NH           0
## NJ           0
## NM           0
## NV           0
## NY           0
## OH           0
## OK           0
## OR           0
## PA           0
## RI           0
## SC           0
## TN           0
## TX           0
## UT           0
## VA           0
## VT           0
## WA           0
## WI           0
## WV           0
##
## with conditional variances for "State"
```

Conversely, the Week parameter only required 20 random effects estimates, and we had 150 measurements from each Week. Below is the random effects estimate for the intercept of each week:

```
ranef(lmer(SRA ~ RP + PSS + Anxiety + Depression +  (1|Week), data=CVA_full))
```

```
## $Week
##        (Intercept)
## Week 1   0.4768978
## Week 2  -1.0420765
## Week 3  -0.6234464
## Week 4   1.1886252
##
## with conditional variances for "Week"
```

The estimated intercept for each week is non-zero. We therefore decided to remove State as a random effect and only included Week. It should be noted that none of the results of the model actually change if State is included. However, given the lack of variance the random effect of State contributed to the model, we believe its removal is justifiable.

We used the R package lme4 to model everyday altruism (Self-Report Altruism scale, SRA in the code) as a function of: Perceived COVID19 threat (risk perception, RP in the code), Stress (perceived stress scale, PSS in the code), Anxiety, and Depression. Additionally, we modelled random intercept and random slope estimates per Week for each of the 4 predictors.

```
m1 <- lmer(SRAz ~ RPz + PSSz + Anxietyz + Depressionz +
             (1+RPz+PSSz+Anxietyz+Depressionz|Week), data=CVA_full)
```

```
## Linear mixed model fit by REML. t-tests use Satterthwaite's method [
## lmerModLmerTest]
## Formula: SRAz ~ RPz + PSSz + Anxietyz + Depressionz + (1 + RPz + PSSz +
##     Anxietyz + Depressionz | Week)
##    Data: CVA_full
##
## REML criterion at convergence: 1655.4
##
## Scaled residuals:
##     Min      1Q  Median      3Q     Max
## -2.8079 -0.6734 -0.0760  0.5869  3.4215
##
## Random effects:
##  Groups   Name        Variance Std.Dev. Corr
##  Week     (Intercept) 0.013550 0.11641
##           RPz         0.002894 0.05380  -0.91
##           PSSz        0.015730 0.12542  -0.21  0.60
##           Anxietyz    0.001844 0.04294  -0.46  0.04 -0.78
##           Depressionz 0.015674 0.12520   0.59 -0.87 -0.91  0.45
##  Residual             0.888206 0.94245
## Number of obs: 600, groups:  Week, 4
##
## Fixed effects:
##              Estimate Std. Error        df t value Pr(>|t|)
## (Intercept) -0.006623   0.069856  3.080415  -0.095  0.93030
## RPz          0.168702   0.049992  5.591384   3.375  0.01666 *
## PSSz        -0.145569   0.084780  3.287907  -1.717  0.17641
## Anxietyz     0.305180   0.055250  9.925111   5.524  0.00026 ***
## Depressionz -0.171918   0.088434  3.580966  -1.944  0.13207
## ---
## Signif. codes:  0 '***' 0.001 '**' 0.01 '*' 0.05 '.' 0.1 ' ' 1
##
## Correlation of Fixed Effects:
##             (Intr) RPz    PSSz   Anxtyz
## RPz         -0.410
## PSSz        -0.129  0.127
## Anxietyz    -0.146 -0.118 -0.255
## Depressionz  0.351 -0.330 -0.757 -0.169
## optimizer (nloptwrap) convergence code: 0 (OK)
## boundary (singular) fit: see ?isSingular
```

Table 1: Table 1

	Estimate	Std. Error	df	t value	Pr(>|t|)	CI_lower	CI_upper
(Intercept)	-0.007	0.070	3.080	-0.095	0.930	-0.144	0.130
RPz	0.169	0.050	5.591	3.375	0.017	0.071	0.267
PSSz	-0.146	0.085	3.288	-1.717	0.176	-0.312	0.021
Anxietyz	0.305	0.055	9.925	5.524	0.000	0.197	0.413
Depressionz	-0.172	0.088	3.581	-1.944	0.132	-0.345	0.001

Perceived Covid19 threat and Anxiety were both statistically significant predictors of SRA. Note that this model (and the subsequent models below) gave us a warning stating we had a boundary (singular) fit. These warnings indicate that one or more variance estimates are very close to zero. While random effects could be removed to eliminate this warning, we felt the inclusion of all random effects was important. Because our models account for a large amount random variance (intercepts and slopes) associated with each week, our model also generalises better to other weeks and individuals (Yarkoni, 2019). While additional random effects can reduce power, they can also reduces the Type-I error rate. Given that with enough data every difference will be statistically significant (Meehl, 1967), and given our sample size, we felt this was a reasonable compromise.

Model 2 - adding gender, age, employment, socio-economic status We also wanted to see how additional fixed effects, not related to our main predictions, explained everyday altruism. To minimize convergence problems, we first selected additional predictors with potential associations with our dependent variable (everyday altruism) and selectively added them to the model (see below). We therefore ran a new model with 4 additional predictors: Age, Gender, Employment, and socioeconomic level. Our goal was to identify predictors associated with SRA at our pre-defined threshold ($p < 0.05$) so that we could add them to the final model.

```
#m2 <- lmer(SRAz ~ Age + Gender2 + Employment + financ +
#              (1+Age + Gender2 + Employment + financ|Week), data=CVA_full)
library(optimx)
#m2 <- lmer(SRAz ~ Age + Gender2 + Employment + financ + (1+Age + Gender2 + Employment + financ|Week),
m2 <- lmer(SRAz ~ Age + Gender2 + Employment + financ + (1+Age + Gender2 + Employment + financ|Week), da
```

```
## Warning in optwrap(optimizer, devfun, getStart(start, rho$pp), lower =
## rho$lower, : convergence code 1 from optimx: none
```

```
## Warning in optimx.check(par, optcfg$ufn, optcfg$ugr, optcfg$uhess, lower, : Parameters or bounds app
##   This can cause poor performance in optimization.
##   It is important for derivative free methods like BOBYQA, UOBYQA, NEWUOA.
```

```
## Warning: Model failed to converge with 1 negative eigenvalue: -4.6e+00
```

```
## Linear mixed model fit by maximum likelihood . t-tests use Satterthwaite's
##   method [lmerModLmerTest]
## Formula: SRAz ~ Age + Gender2 + Employment + financ + (1 + Age + Gender2 +
##     Employment + financ | Week)
##    Data: CVA_full
## Control: lmerControl(optimizer = "optimx", optCtrl = list(method = "L-BFGS-B"))
##
##      AIC      BIC   logLik deviance df.resid
##   1709.9   1951.7   -799.9   1599.9      545
```

```
##
## Scaled residuals:
##     Min      1Q  Median      3Q     Max
## -2.7900 -0.6892 -0.0337  0.5940  3.7898
##
## Random effects:
##  Groups   Name                          Variance  Std.Dev. Corr
##  Week     (Intercept)                   4.701e-02 0.21681
##           Age                           6.866e-06 0.00262  -1.00
##           Gender2Male                   3.293e-02 0.18146  -0.43  0.43
##           Gender2Other                  1.890e-01 0.43473   0.44 -0.44  0.18
##           EmploymentStudent             2.592e-02 0.16101  -0.80  0.80  0.30
##           EmploymentUnemployed          3.992e-02 0.19980   0.52 -0.52 -0.97
##           financJust meet basic expenses  1.040e-03 0.03225   0.34 -0.34 -0.63
##           financLive comfortably        3.898e-02 0.19743  -0.99  0.99  0.47
##           financMeet needs with some left 1.402e-02 0.11841   0.30 -0.30 -0.34
##  Residual                               8.236e-01 0.90754
##
##
##
##
##
##  -0.85
##   0.05 -0.50
##   0.54 -0.71  0.78
##  -0.53  0.87 -0.59 -0.48
##   0.77 -0.78  0.54  0.94 -0.44
##
## Number of obs: 600, groups:  Week, 4
##
## Fixed effects:
##                                  Estimate Std. Error        df t value Pr(>|t|)
## (Intercept)                     -0.548738   0.214625  6.714093  -2.557  0.03909
## Age                              0.029075   0.004145  4.568322   7.014  0.00131
## Gender2Male                      0.003109   0.118859  4.071964   0.026  0.98037
## Gender2Other                     0.170219   0.355779  4.680605   0.478  0.65384
## EmploymentStudent               -0.204300   0.135196  3.446626  -1.511  0.21633
## EmploymentUnemployed            -0.445272   0.137646  4.823576  -3.235  0.02430
## financJust meet basic expenses  -0.293573   0.141673 90.222409  -2.072  0.04110
## financLive comfortably          -0.064411   0.184295  5.406895  -0.349  0.73992
## financMeet needs with some left -0.278842   0.153054  4.909843  -1.822  0.12917
##
## (Intercept)                     *
## Age                             **
## Gender2Male
## Gender2Other
## EmploymentStudent
## EmploymentUnemployed            *
## financJust meet basic expenses  *
## financLive comfortably
## financMeet needs with some left
## ---
## Signif. codes:  0 '***' 0.001 '**' 0.01 '*' 0.05 '.' 0.1 ' ' 1
##
```

```
## Correlation of Fixed Effects:
##             (Intr) Age    Gndr2M Gndr2O EmplyS EmplyU fnJmbe fnncLc
## Age         -0.696
## Gender2Male -0.260  0.096
## Gender2Othr  0.082 -0.044  0.145
## EmplymntStd -0.532  0.451  0.123 -0.303
## EmplymntUnm  0.070 -0.216 -0.500 -0.025 -0.107
## fnncJstmtbe -0.504 -0.046 -0.045  0.061 -0.023  0.175
## fnncLvcmfrt -0.663  0.133  0.165 -0.199  0.267 -0.107  0.579
## fnncMtndwsl -0.431 -0.084 -0.111  0.180 -0.125  0.299  0.779  0.484
## optimizer (optimx) convergence code: 0 (OK)
## boundary (singular) fit: see ?isSingular
## Parameters or bounds appear to have different scalings.
##   This can cause poor performance in optimization.
##   It is important for derivative free methods like BOBYQA, UOBYQA, NEWUOA.
```

This model, however, did not converge. Convergence issues can be caused by a number of factors, and we decided to explore ways to get this model working properly.

In an attempt to correct convergence issues, we centered age in the model (it was the only variable that could be centered). This can sometimes help when a model does not converge, and is a common strategy when first attempting to deal with non-convergence.

```
m2.2 <- lmer(SRAz ~ agez + Gender2 + Employment + financ +
               (1+agez + Gender2 + Employment + financ|Week), data=CVA_full, control = lmerControl(opti
```

```
## Linear mixed model fit by REML. t-tests use Satterthwaite's method [
## lmerModLmerTest]
## Formula: SRAz ~ agez + Gender2 + Employment + financ + (1 + agez + Gender2 +
##     Employment + financ | Week)
##    Data: CVA_full
## Control: lmerControl(optimizer = "bobyqa", optCtrl = list(maxfun = 1e+05))
##
## REML criterion at convergence: 1622.6
##
## Scaled residuals:
##     Min      1Q  Median      3Q     Max
## -2.7888 -0.6911 -0.0317  0.5744  3.7503
##
## Random effects:
##  Groups   Name                         Variance Std.Dev. Corr
##  Week     (Intercept)                  0.013595 0.11660
##           agez                         0.002447 0.04947  -0.39
##           Gender2Male                  0.048456 0.22013  -0.52  0.14
##           Gender2Other                 0.305692 0.55289   0.07 -0.85  0.41
##           EmploymentStudent            0.037476 0.19359  -0.32  1.00  0.10
##           EmploymentUnemployed         0.055776 0.23617   0.50 -0.51 -0.91
##           financJust meet basic expenses 0.007528 0.08676   0.52 -0.99 -0.24
##           financLive comfortably       0.035106 0.18737  -0.95  0.66  0.51
##           financMeet needs with some left 0.035167 0.18753   0.12 -0.94 -0.16
##  Residual                              0.831632 0.91194
##
##
##
```

```
##
##
##  -0.86
##  -0.02 -0.49
##   0.78 -0.98  0.58
##  -0.33  0.60 -0.62 -0.76
##   0.79 -0.96  0.55  0.90 -0.43
##
## Number of obs: 600, groups:  Week, 4
##
## Fixed effects:
##                                  Estimate Std. Error        df t value Pr(>|t|)
## (Intercept)                      0.352754   0.152403  9.689231   2.315  0.04395
## agez                             0.310576   0.048953  6.131630   6.344  0.00066
## Gender2Male                      0.003842   0.134437  3.210545   0.029  0.97889
## Gender2Other                     0.210135   0.396677  2.865031   0.530  0.63458
## EmploymentStudent               -0.199281   0.145900  4.282029  -1.366  0.23933
## EmploymentUnemployed            -0.447614   0.151739  3.423128  -2.950  0.05095
## financJust meet basic expenses  -0.287466   0.148111 18.811540  -1.941  0.06741
## financLive comfortably          -0.066790   0.182454  6.299677  -0.366  0.72630
## financMeet needs with some left -0.276832   0.170033  5.675809  -1.628  0.15744
##
## (Intercept)                     *
## agez                            ***
## Gender2Male
## Gender2Other
## EmploymentStudent
## EmploymentUnemployed            .
## financJust meet basic expenses  .
## financLive comfortably
## financMeet needs with some left
## ---
## Signif. codes:  0 '***' 0.001 '**' 0.01 '*' 0.05 '.' 0.1 ' ' 1
##
## Correlation of Fixed Effects:
##             (Intr) agez   Gndr2M Gndr2O EmplyS EmplyU fnJmbe fnncLc
## agez        -0.067
## Gender2Male -0.286  0.051
## Gender2Othr -0.022 -0.261  0.284
## EmplymntStd -0.227  0.589  0.046 -0.392
## EmplymntUnm -0.084 -0.281 -0.551 -0.049 -0.158
## fnncJstmtbe -0.684 -0.176 -0.048  0.180 -0.167  0.230
## fnncLvcmfrt -0.785  0.140  0.188 -0.138  0.194 -0.132  0.480
## fnncMtndwsl -0.639 -0.303 -0.080  0.302 -0.306  0.356  0.788  0.408
## optimizer (bobyqa) convergence code: 0 (OK)
## boundary (singular) fit: see ?isSingular
```

The model now converges, and we can see that age is the only clear significant predictor of SRA. However, employment appeared to be a potentially relevant predictor (marginally significant association with SRA), so we included it in the final model.

Model 3 - Adding Age and Employment to Model 1 We added age and employment to the initial model. However, the updated model no longer converged. Another common strategy to aid convergence is

to remove the most complex random effects (i.e. random slopes) iteratively from the model. Because the random effects of age accounted for the least amount of variance in the new model, we removed the random slope estimate of age and re-ran the model.

```
m3 <- lmer(SRAz ~ RPz + PSSz + Anxietyz + Depressionz + agez + Employment +
             (1+RPz+PSSz+Anxietyz+Depressionz + Employment|Week), data=CVA_full)
```

```
## Linear mixed model fit by REML. t-tests use Satterthwaite's method [
## lmerModLmerTest]
## Formula: SRAz ~ RPz + PSSz + Anxietyz + Depressionz + agez + Employment +
##     (1 + RPz + PSSz + Anxietyz + Depressionz + Employment | Week)
##    Data: CVA_full
##
## REML criterion at convergence: 1586.2
##
## Scaled residuals:
##     Min      1Q  Median      3Q     Max
## -2.6035 -0.6475 -0.0473  0.6177  3.4292
##
## Random effects:
##  Groups   Name                 Variance Std.Dev. Corr
##  Week     (Intercept)          0.010039 0.10020
##           RPz                  0.003969 0.06300  -1.00
##           PSSz                 0.004415 0.06645  -0.25  0.29
##           Anxietyz             0.001894 0.04352  -0.48  0.44 -0.73
##           Depressionz          0.005381 0.07336   0.63 -0.66 -0.91  0.38
##           EmploymentStudent    0.012283 0.11083  -0.98  0.97  0.04  0.65 -0.45
##           EmploymentUnemployed 0.056835 0.23840   0.60 -0.56  0.63 -0.99 -0.25
##  Residual                      0.779101 0.88267
##
##
##
##
##
##
##
##  -0.75
##
## Number of obs: 600, groups:  Week, 4
##
## Fixed effects:
##                       Estimate Std. Error        df t value Pr(>|t|)
## (Intercept)            0.11875    0.06987   3.60042   1.700 0.172302
## RPz                    0.15612    0.05061   4.69036   3.085 0.029731 *
## PSSz                  -0.08360    0.06326   5.32891  -1.321 0.240229
## Anxietyz               0.29570    0.05246   9.24913   5.637 0.000289 ***
## Depressionz           -0.13257    0.06907   5.16353  -1.919 0.111196
## agez                   0.31386    0.04191 577.04770   7.489 2.61e-13 ***
## EmploymentStudent     -0.12018    0.11872   8.83264  -1.012 0.338332
## EmploymentUnemployed  -0.41598    0.14940   3.27288  -2.784 0.062258 .
## ---
## Signif. codes:  0 '***' 0.001 '**' 0.01 '*' 0.05 '.' 0.1 ' ' 1
##
## Correlation of Fixed Effects:
```

```
##             (Intr) RPz    PSSz   Anxtyz Dprssn agez   EmplyS
## RPz         -0.448
## PSSz        -0.049 -0.033
## Anxietyz    -0.157 -0.007 -0.203
## Depressionz  0.241 -0.217 -0.666 -0.258
## agez        -0.064 -0.044  0.062  0.007  0.073
## EmplymntStd -0.636  0.281 -0.025  0.160 -0.089  0.353
## EmplymntUnm  0.120 -0.281  0.201 -0.319 -0.120 -0.101 -0.176
## optimizer (nloptwrap) convergence code: 0 (OK)
## boundary (singular) fit: see ?isSingular
```

Table 2: Table 2

	Estimate	Std. Error	df	t value	Pr(>|t|)	CI_lower	CI_upper
(Intercept)	0.119	0.070	3.600	1.700	0.172	-0.018	0.256
RPz	0.156	0.051	4.690	3.085	0.030	0.057	0.255
PSSz	-0.084	0.063	5.329	-1.321	0.240	-0.208	0.040
Anxietyz	0.296	0.052	9.249	5.637	0.000	0.193	0.399
Depressionz	-0.133	0.069	5.164	-1.919	0.111	-0.268	0.003
agez	0.314	0.042	577.048	7.489	0.000	0.232	0.396
EmploymentStudent	-0.120	0.119	8.833	-1.012	0.338	-0.353	0.113
EmploymentUnemployed	-0.416	0.149	3.273	-2.784	0.062	-0.709	-0.123

With the random slopes associated with age removed, the new model converged. We then assessed whether the full model (perceived covid-19 threat, anxiety, stress, depression, age and employment) was prefered to the original model (without age and employment) using a model comparison approach. Results suggested a better fit of the full model.

```
## Data: CVA_full
## Models:
## m1: SRAz ~ RPz + PSSz + Anxietyz + Depressionz + (1 + RPz + PSSz +
## m1:     Anxietyz + Depressionz | Week)
## m3: SRAz ~ RPz + PSSz + Anxietyz + Depressionz + agez + Employment +
## m3:     (1 + RPz + PSSz + Anxietyz + Depressionz + Employment | Week)
##    npar    AIC    BIC  logLik deviance  Chisq Df Pr(>Chisq)
## m1   21 1677.5 1769.8 -817.74   1635.5
## m3   37 1629.6 1792.3 -777.80   1555.6 79.895 16  1.738e-10 ***
## ---
## Signif. codes:  0 '***' 0.001 '**' 0.01 '*' 0.05 '.' 0.1 ' ' 1
```

For the sake of transparency, we also present here the results of the model if all predictors had been added simultaneously.

```
m_all <- lmer(SRAz ~ RPz + PSSz + Anxietyz + Depressionz +Age + Gender2 + Employment + financ +
             (1+Age + Gender2 + Employment + financ+ RPz + PSSz + Anxietyz + Depressionz|Week), data=CVA
```

```
## Warning: Model failed to converge with 3 negative eigenvalues: -1.7e-01 -7.0e+00
## -1.1e+01
```

```
## Linear mixed model fit by REML. t-tests use Satterthwaite's method [
## lmerModLmerTest]
## Formula: SRAz ~ RPz + PSSz + Anxietyz + Depressionz + Age + Gender2 +
##     Employment + financ + (1 + Age + Gender2 + Employment + financ +
##     RPz + PSSz + Anxietyz + Depressionz | Week)
##    Data: CVA_full
##
## REML criterion at convergence: 1577.1
##
## Scaled residuals:
##      Min       1Q   Median       3Q      Max
## -2.65502 -0.66308 -0.04874  0.59609  3.11533
##
```

```
## Random effects:
##  Groups   Name                             Variance  Std.Dev. Corr
##  Week     (Intercept)                      6.481e-02 0.254570
##           Age                              3.150e-06 0.001775 -1.00
##           Gender2Male                      4.838e-02 0.219953 -1.00  1.00
##           Gender2Other                     2.772e-01 0.526492 -0.24  0.24  0.24
##           EmploymentStudent                1.933e-02 0.139041 -0.34  0.34  0.34
##           EmploymentUnemployed             3.853e-02 0.196299  0.92 -0.92 -0.92
##           financJust meet basic expenses   1.236e-02 0.111176 -0.31  0.31  0.32
##           financLive comfortably           6.374e-02 0.252478 -0.98  0.98  0.98
##           financMeet needs with some left  4.185e-02 0.204581 -0.25  0.25  0.25
##           RPz                              5.927e-03 0.076984 -0.43  0.43  0.43
##           PSSz                             2.383e-03 0.048817  0.07 -0.07 -0.05
##           Anxietyz                         2.868e-03 0.053558 -0.98  0.98  0.98
##           Depressionz                      5.428e-03 0.073675 -0.27  0.27  0.27
##  Residual                                  7.459e-01 0.863643
##
##
##
##
##
##  -0.83
##   0.15 -0.68
##   0.81 -0.62  0.02
##   0.08  0.49 -0.97  0.15
##   1.00 -0.82  0.14  0.86  0.09
##  -0.77  0.99 -0.75 -0.60  0.57 -0.76
##  -0.89  0.81 -0.28 -0.45  0.06 -0.85  0.74
##   0.09  0.48 -0.96  0.12  0.99  0.09  0.57  0.02
##   0.99 -0.80  0.12  0.75  0.12  0.98 -0.73 -0.92  0.13
##
## Number of obs: 600, groups:  Week, 4
##
## Fixed effects:
##                                  Estimate Std. Error        df t value Pr(>|t|)
## (Intercept)                     -0.647356   0.223727  5.092804  -2.894  0.03332
## RPz                              0.152723   0.054706  3.694701   2.792  0.05387
## PSSz                            -0.091500   0.059584  7.483571  -1.536  0.16576
## Anxietyz                         0.298247   0.053963  6.647110   5.527  0.00105
## Depressionz                     -0.130219   0.069302  5.688304  -1.879  0.11198
## Age                              0.028712   0.003927 24.697396   7.311 1.26e-07
## Gender2Male                      0.045237   0.133405  3.133222   0.339  0.75600
## Gender2Other                     0.274359   0.376707  3.534395   0.728  0.51172
## EmploymentStudent               -0.164586   0.124928  5.984934  -1.317  0.23587
## EmploymentUnemployed            -0.433552   0.133754  3.547363  -3.241  0.03762
## financJust meet basic expenses  -0.240331   0.147235 13.274486  -1.632  0.12610
## financLive comfortably           0.023325   0.199034  4.482359   0.117  0.91177
## financMeet needs with some left -0.180514   0.172188 10.972503  -1.048  0.31702
##
## (Intercept)                     *
## RPz                             .
## PSSz
## Anxietyz                        **
## Depressionz
```

```
## Age                              ***
## Gender2Male
## Gender2Other
## EmploymentStudent
## EmploymentUnemployed             *
## financJust meet basic expenses
## financLive comfortably
## financMeet needs with some left
## ---
## Signif. codes:  0 '***' 0.001 '**' 0.01 '*' 0.05 '.' 0.1 ' ' 1
## optimizer (nloptwrap) convergence code: 0 (OK)
## boundary (singular) fit: see ?isSingular
```

The full model failed to converge, but the results were in the same direction as with the other models.

Testing assumptions

1. Homoskedasticity: Residuals are randomly distributed around the regression line.
2. Normality: residuals are normally distributed as can be assessed using either a histogram or qq-plot.
3. Random effects residuals: The random effects estimates should also be normally distributed around zero

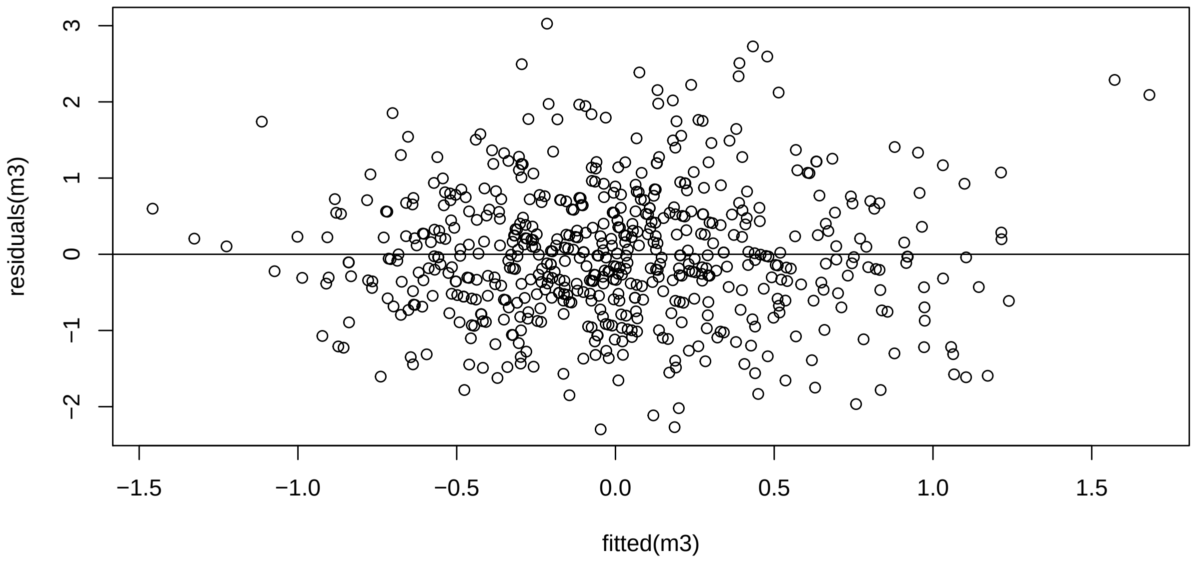

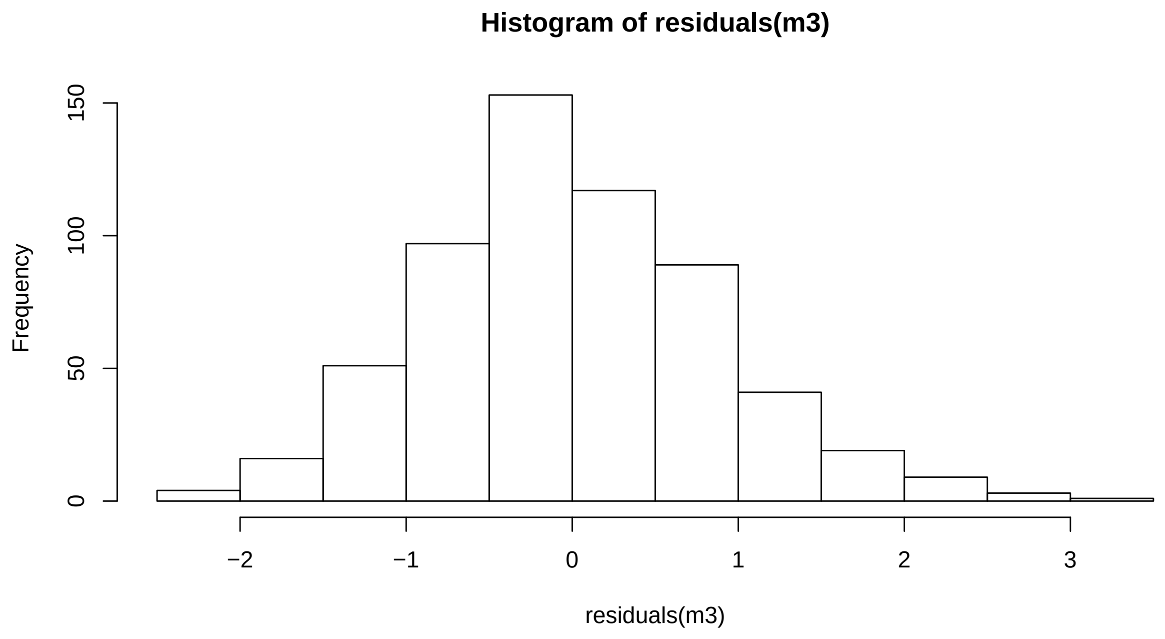
Histogram of residuals(m3)
Frequency
0
50
100
150
−2
−1
0
1
2
3
residuals(m3)

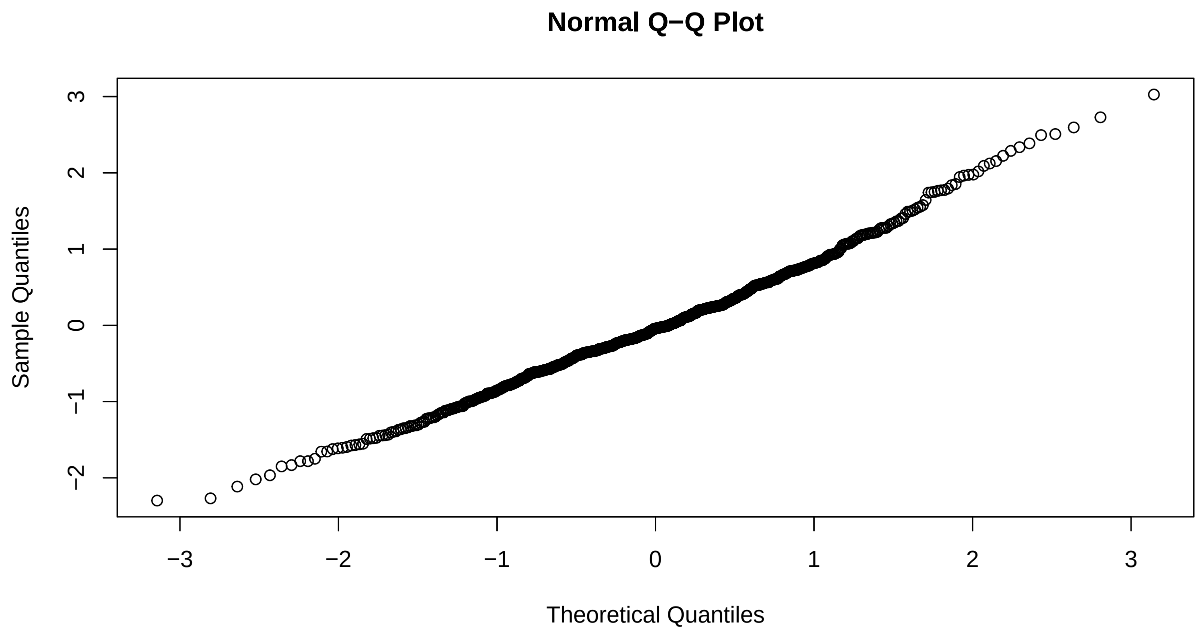
Normal Q−Q Plot
Sample Quantiles
−2
−1
0
1
2
3
−3
−2
−1
0
1
2
3
Theoretical Quantiles

```
## $Week
```

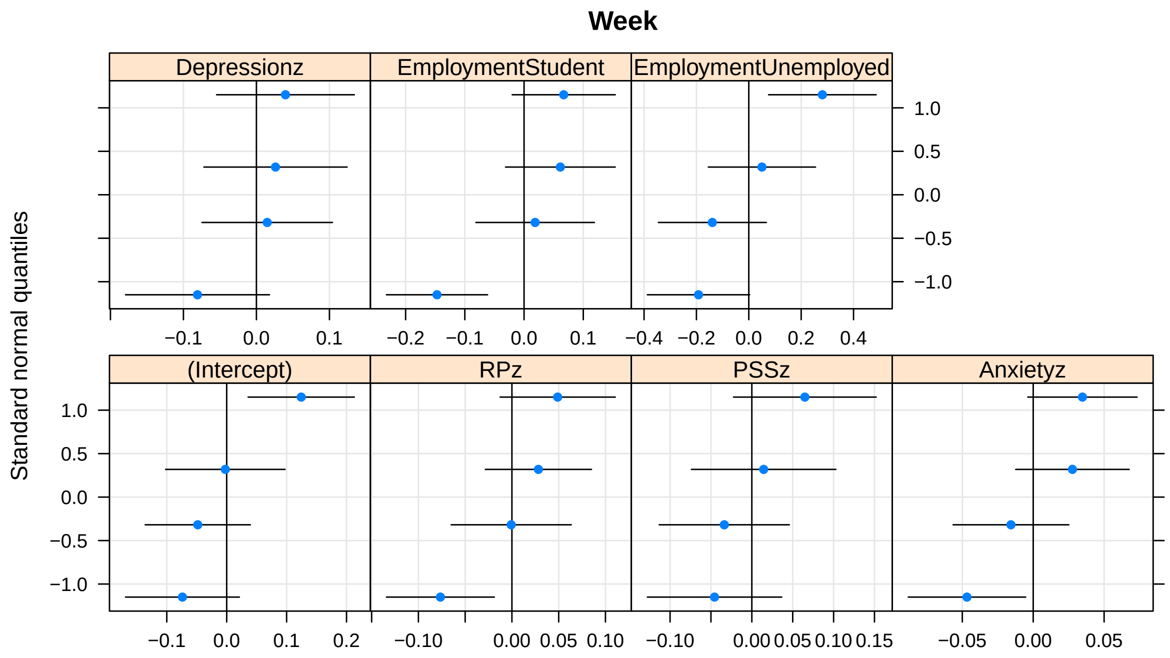

2. Modelling everyday altruism within each week We tested our model in each independent week to examined whether the observed associations between self-reported altruism, and perceived covid-19 risk and anxiety held over time (rather than accounting for that by modelling week as a random effect, as done in the previous analysis).

When modelling each week separately, the model used above had to be simplified to provide reliable estimates and avoid convergence issues, given the reduction in the number of observations (600 to 150 participants). Specifically, we removed all random effects of by all fixed effects, because we were no longer using Week as random effect (SRAz ~ RPz + Anxietyz + Depressionz+ PSSz + agez + Employment). Results (Table 3) show anxiety was consistently associated with increased everyday altruism within each week.

3. State variation in altruism and COVID-19 risk perception Our main analysis using accounted for variation introduced by variability between weeks of data collection. Presumably, there could be variability associated with each location we collected data from, even if this variability was not enough to influence the inclusion of state into our model. However, we understand state variation could be of interest to some readers. We thus include here some exploratory plots to help readers visualise our data in reference to the state.

The first plot presented illustrates the number of cases per State in the 4 weeks of data collection.

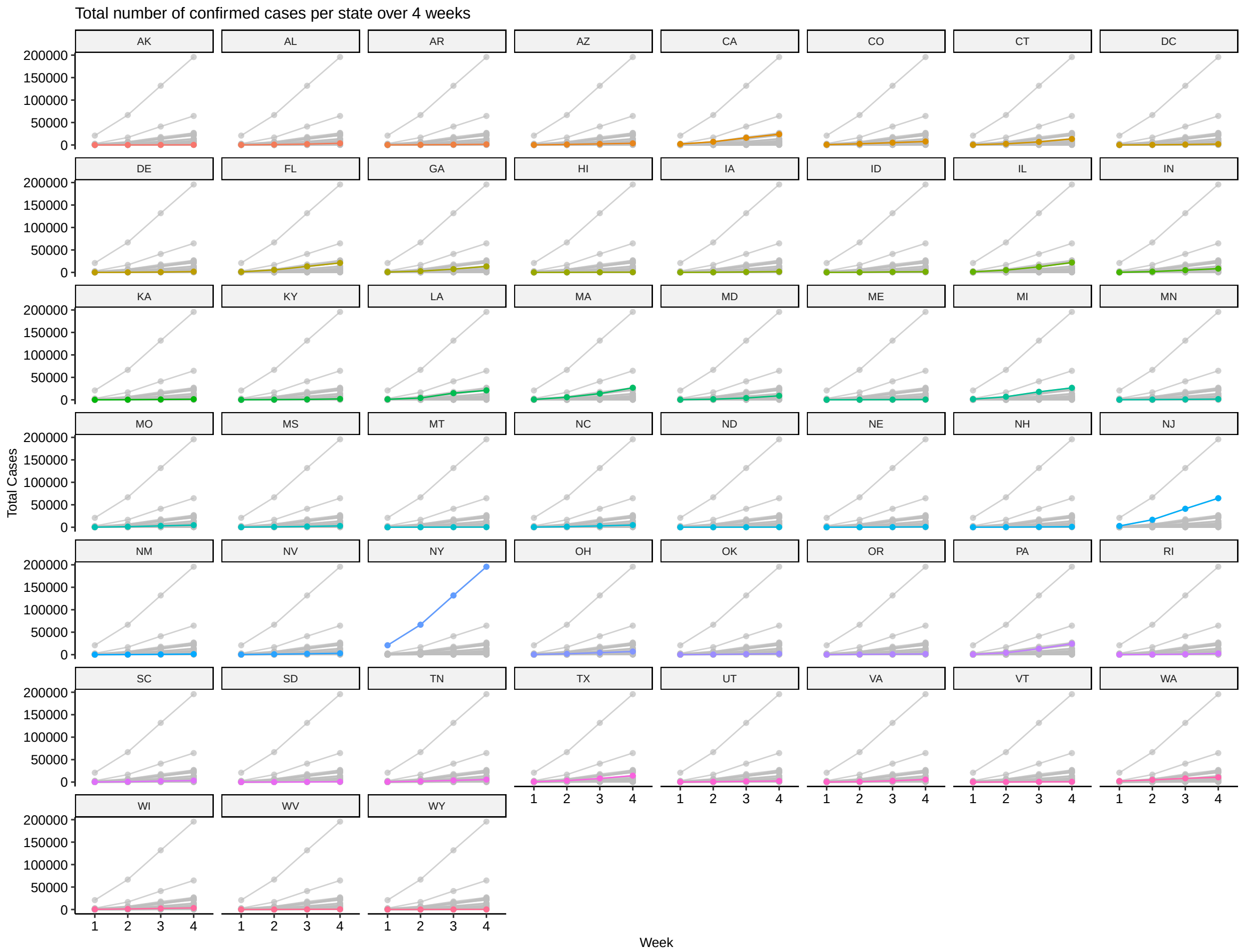

One question we can anticipate from readers is that, due to the great variability in severity of the pandemic in different states (e.g., NY versus Alaska), it may be that the key experimental effects could vary greatly between states. However, as explained above, most states in our sample had a reduced number of cases, making variation due to State a likely noisy effect. For descriptive purposes, here we selected states that we collected 20 or more observations from. Below, we plot results from those states for variables of interest in our study, namely Perceived Covid19 threat (RP), Self-Reported Altruism, and Total Confirmed COVID cases.

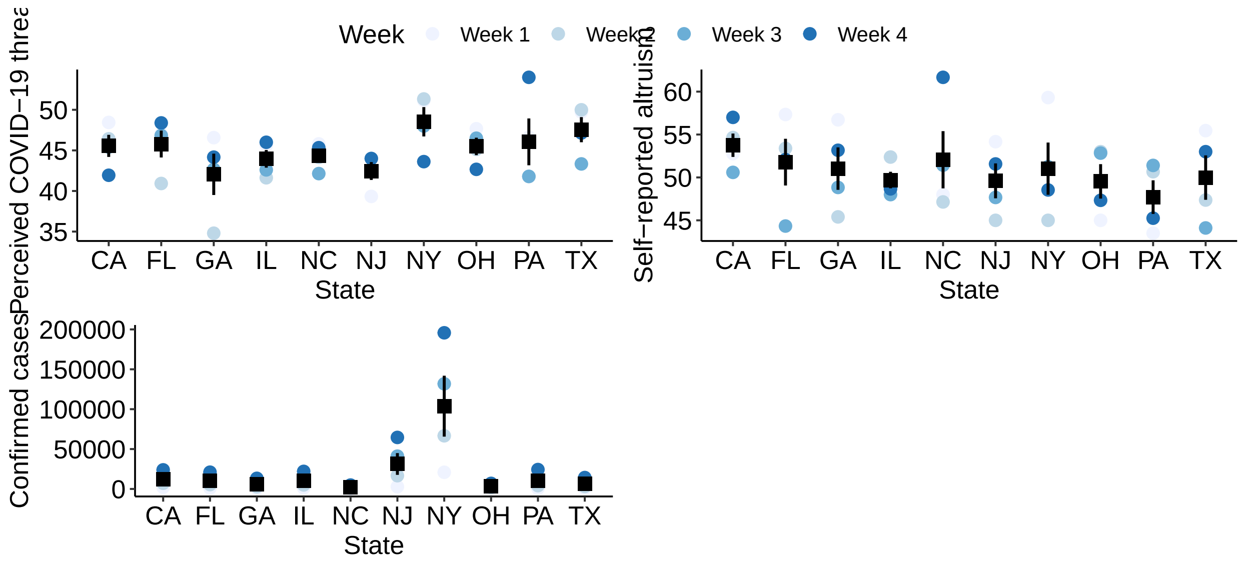

The plot above depicts how Perceived Covid19 threat, self-reported altruism and Number of confirmed cases evolved over 4 weeks of data collection, for each of the states with N>20. As can be seen in the plot, NJ and NY were the States that had the largest and most obvious increase in number of cases. For Perceived COVID19 threat and self-reported altruism, no week-related pattern seems to emerge across all the states included. We next plotted Perceived Covid19 threat and Self-Reported Altruism from those 2 States below, to illustrate that even in States with a clear rise in cases, these measures do not seem to meaningfully change over the 4 weeks.

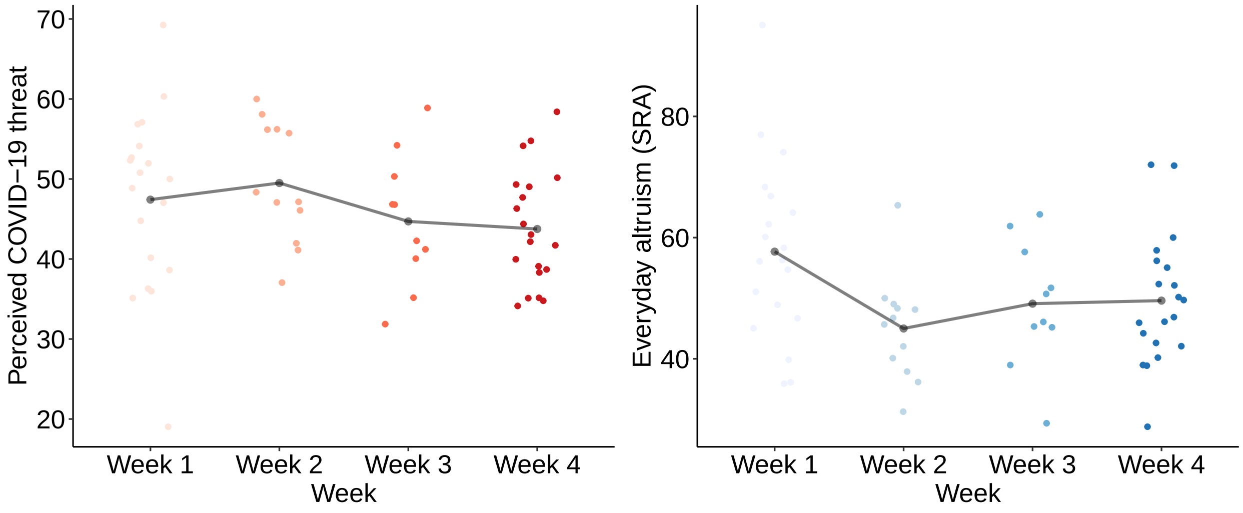

The ANOVAs below show no significant changes in Perceived COVID-19 threat in NY/NJ over the 4 weeks. For SRA, there is an apparent decrease from week 1 to week 2, but consistent pattern emerged. Note that this analysis is purely exploratory, as the sample size per week is very small (week 1: n=19, week 2: n = 12, week 3: n=10; week 4: n = 20).

```
## # A tibble: 4 x 2
##   Week    `n()`
##   <fct>   <int>
## 1 Week 1     19
## 2 Week 2     12
## 3 Week 3     10
```

```
## 4 Week 4    20

##
##  Pairwise comparisons using t tests with pooled SD
##
## data:  NY_NJ$SRA and NY_NJ$Week
##
##        Week 1 Week 2 Week 3
## Week 2 0.031  -      -
## Week 3 0.274  0.874  -
## Week 4 0.186  0.874  0.913
##
## P value adjustment method: holm
```

Simulation-based power analysis for random effects model

Because our study was constrained by time and resources, we did not conduct a formal power analysis prior to data collection and analysis. Power analyses can still be useful after a study is completed as they can reveal how much power a study theoretically had to detect a range of effect sizes of interest.

We opted to model a simple power function at fixed (i.e. discrete steps) effect sizes for the 4 key predictors (Perceived Covid-19 Threat, RP; Anxiety; Stress, PSS; Depression) in our model by simulating a dataset. We first simulated standardised questionnaire data to match how it might reasonably be generated from individuals randomly sampled from the population. Thus, we simulated data with 600 observations for 4 parameter by drawing values randomly from a normal distribution with a mean of zero, standard deviation of 1, and a correlation between each observations parameter estimate of 0.4. Additionally, each observation was assigned a week, and a random variance component was included with each observation by drawing 4 values from a normal distribution with a mean of zero and a standard deviation of 1. We caculated an SRA score for each observation in the model by multiplying a fixed parameter estimate to each simulated questionnaire score and then adding together the linear combination of these values (along with weekly random variance and a randomly drawn error term). The data structure for the first 5 simulated observations (i.e. participants) can be seen below.

```
##          RPs          PSSs    Anxietys Depressions       SRAs  Week
## 1 -0.8072558 -1.3779339987 -0.25845530  -2.1452341  3.5373848 Week1
## 2 -0.5469162  0.8939096893  1.02162080  -0.3731908  3.6227388 Week1
## 3 -0.5543976  1.0565235744 -0.95868067   1.0032606  0.2742031 Week1
## 4 -0.7192858 -0.0003644981 -2.87045860   0.3082502 -0.1373379 Week1
## 5  0.8367482  0.7873318519  2.45475624   0.4479177  3.8014265 Week1
## 6  0.6320206 -0.2752098637 -0.03487023  -0.4420508  1.0225829 Week1
```

Fitting the model

The simulated data were fit to the same model used in the study. We fixed all slopes at 0.3 in the simulation, and thus, we expected their subsequent random effects model estimates to roughly match the fixed 0.3 terms. As can be seen below, these assumptions are roughly met in our model output.

```
## Linear mixed model fit by REML. t-tests use Satterthwaite's method [
## lmerModLmerTest]
## Formula: SRAs ~ RPs + PSSs + Anxietys + Depressions + (1 + RPs + PSSs +
##     Anxietys + Depressions | Week)
##    Data: dat
```

```
##
## REML criterion at convergence: 1711.9
##
## Scaled residuals:
##     Min      1Q  Median      3Q     Max
## -2.4346 -0.6656 -0.0601  0.6538  3.4588
##
## Random effects:
##  Groups   Name        Variance  Std.Dev. Corr
##  Week     (Intercept) 0.1786488 0.42267
##           RPs         0.0026973 0.05194  -0.47
##           PSSs        0.0003819 0.01954  -0.63 -0.39
##           Anxietys    0.0036022 0.06002   0.29 -0.98  0.56
##           Depressions 0.0029689 0.05449   0.70 -0.96  0.11  0.89
##  Residual             0.9670527 0.98339
## Number of obs: 600, groups:  Week, 4
##
## Fixed effects:
##             Estimate Std. Error       df t value Pr(>|t|)
## (Intercept)  0.87185    0.21515  2.99632   4.052 0.027135 *
## RPs          0.36084    0.05336  5.89859   6.763 0.000548 ***
## PSSs         0.28355    0.04880 34.80066   5.811  1.4e-06 ***
## Anxietys     0.32812    0.05653  4.17892   5.804 0.003825 **
## Depressions -0.37704    0.05277  5.23475  -7.145 0.000687 ***
## ---
## Signif. codes:  0 '***' 0.001 '**' 0.01 '*' 0.05 '.' 0.1 ' ' 1
##
## Correlation of Fixed Effects:
##             (Intr) RPs    PSSs   Anxtys
## RPs         -0.214
## PSSs        -0.137 -0.245
## Anxietys     0.152 -0.427 -0.134
## Depressions  0.354 -0.374 -0.152  0.070
## optimizer (nloptwrap) convergence code: 0 (OK)
## boundary (singular) fit: see ?isSingular
```

Running the power simulations

To assess how much power our study had to detect different effect sizes of interest, we ran a power simulation. Here we asked how much power we would have to detect a wide range of effect sizes for each parameter in our model. We thus simulated "power" for 9 effect sizes (0.1, 0.2, 0.3, 0.4, 0.5, 0.6, 0.7, 0.8, 0.9) for each of the 4 parameters. Each simulation started by first simulating new SRA values based on the current fixed effect size, refitting the model to the new simulated SRA values, and running the statistical model again. This was repeated 1000 times for each effect size and each parameter for a total of 36000 simulation.

Power curve

Plotted below are approximated power curves, each showing the proportion of simulations that gave a significant result for each parameter at each fixed effect size. Because power is a contiuous function, rather than a single value, the plot can be analysed to assess how much power the current study would have to detect each effect size, conditional on our random effects model. Each colored line in each plot represents the estimated power for 1 predictor at each fixed effect size, with grey lines showing the other power curves of the other predictors to allow quick comparisons. As an example of how to interpret the graph below, the

power simulation indicates that the sampling procedure for the current study has between 88% and 94% power to detect a 0.3 effect size for each parameter. This logic can be extended to any other fixed effect sizes.

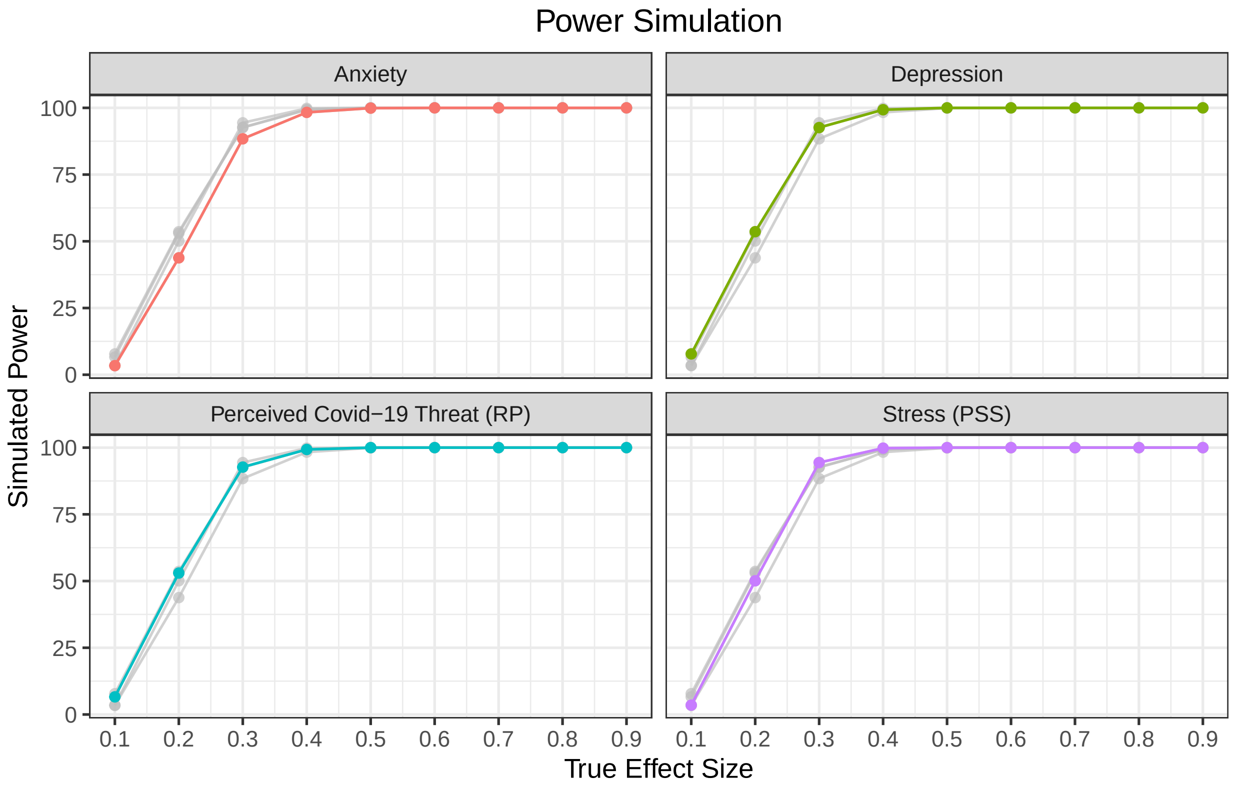

Revisions

Multicollinearity

```
# Kappa function.
kappa.mer <- function (fit,
                       scale = TRUE, center = FALSE,
                       add.intercept = TRUE,
                       exact = FALSE) {
  X <- fit@pp$X
  nam <- names(fixef(fit))

  ## exclude intercepts
  nrp <- sum(1 * (nam == "(Intercept)"))
  if (nrp > 0) {
    X <- X[, -(1:nrp), drop = FALSE]
    nam <- nam[-(1:nrp)]
  }

  if (add.intercept) {
    X <- cbind(rep(1), scale(X, scale = scale, center = center))
    kappa(X, exact = exact)
```

```
  } else {
    kappa(scale(X, scale = scale, center = scale), exact = exact)
  }
}
```

First, we calculated kappa for the model with only the main predictors.

```
kappa.mer(m1) # anything below 10 is reasonable multicollinearity
```

```
## [1] 3.65742
```

Second, kappa for the full model (with demographic predictors) was calculated.

```
kappa.mer(m3) # anything below 10 is reasonable multicollinearity
```

```
## [1] 3.638031
```

Next, we investigated VIF for the model with only main predictors. All predictors have a low correlation.

```
x1 <- check_collinearity(m1)
x1 #
```

```
## # Check for Multicollinearity
##
## Low Correlation
##
##         Term  VIF Increased SE Tolerance
##          RPz 1.37         1.17      0.73
##         PSSz 4.27         2.07      0.23
##     Anxietyz 1.87         1.37      0.54
##  Depressionz 4.66         2.16      0.21
```

```
plot(x1)
```

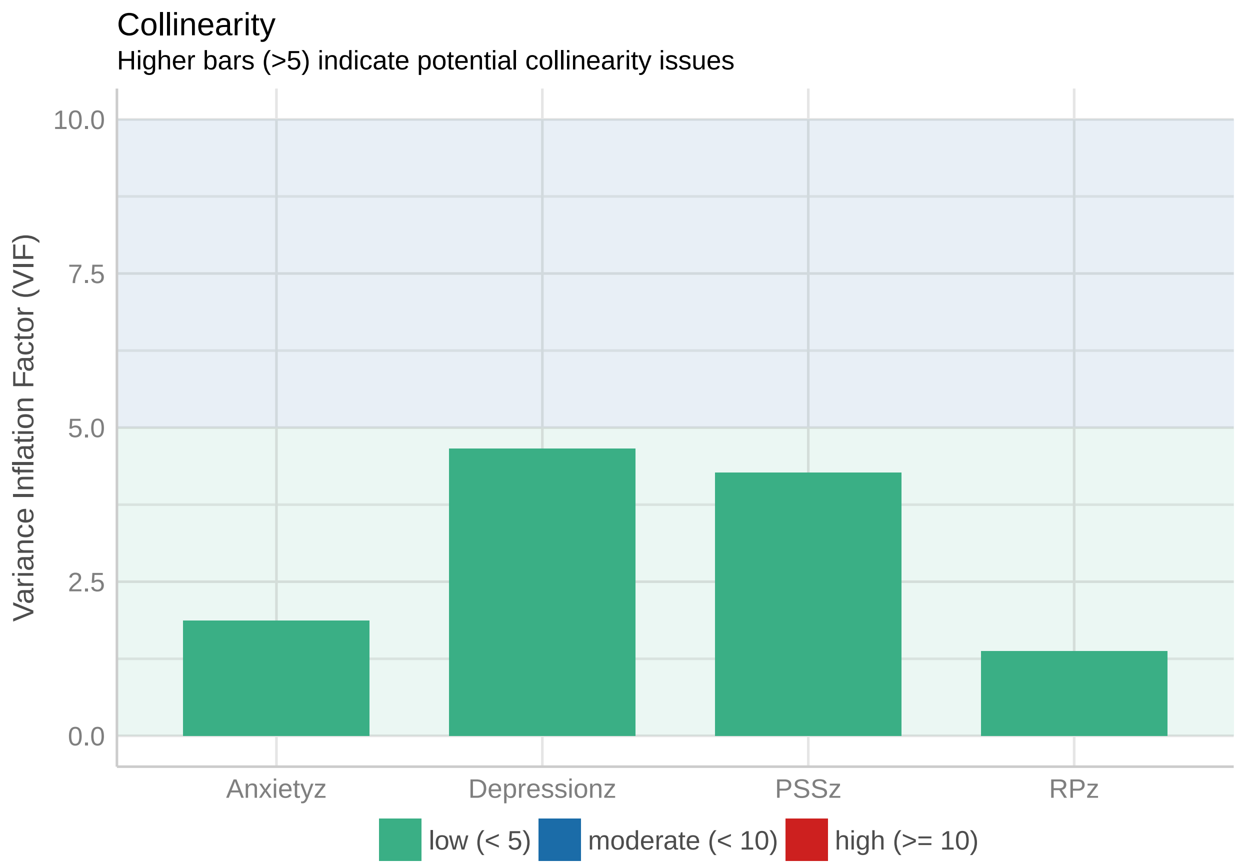

Finally, here is the VIF for the full model. Again, all predictors have a low correlation.

```
x2 <- check_collinearity(m3)
x2 #
```

```
## # Check for Multicollinearity
##
## Low Correlation
##
##         Term  VIF Increased SE Tolerance
##          RPz 1.51         1.23      0.66
##         PSSz 2.93         1.71      0.34
##     Anxietyz 1.94         1.39      0.52
##  Depressionz 3.42         1.85      0.29
##         agez 1.23         1.11      0.81
##   Employment 1.79         1.34      0.56
```

```
plot(x2)
```

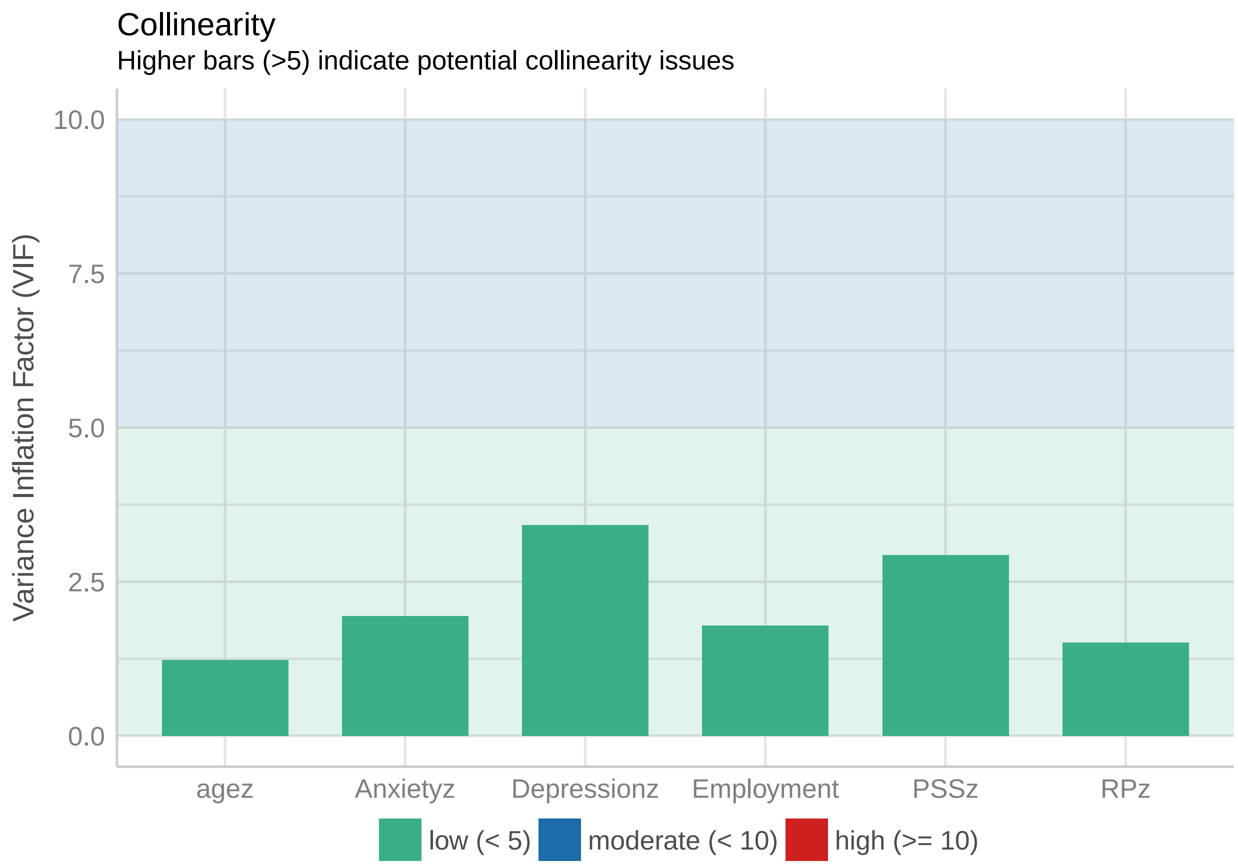

Checking The Effect Of Depression On The Model.

Removing depression from the model with only main predictors did not change the significance of the anxiety or RP coefficients. Additionally,PSS is negatively associated with Altruism.

```
m1_NoDepression <- lmer(SRAz ~ RPz + PSSz + Anxietyz +
             (1+RPz+PSSz+Anxietyz|Week), data=CVA_full)
```

```
## Linear mixed model fit by REML. t-tests use Satterthwaite's method [
## lmerModLmerTest]
## Formula: SRAz ~ RPz + PSSz + Anxietyz + (1 + RPz + PSSz + Anxietyz | Week)
##    Data: CVA_full
##
## REML criterion at convergence: 1661.8
##
## Scaled residuals:
##     Min      1Q  Median      3Q     Max
## -2.6862 -0.6665 -0.0630  0.5605  3.4200
##
## Random effects:
##  Groups   Name        Variance  Std.Dev. Corr
##  Week     (Intercept) 0.0116516 0.10794
##           RPz         0.0019813 0.04451  -1.00
##           PSSz        0.0016823 0.04102   0.36 -0.36
##           Anxietyz    0.0008114 0.02849  -0.13  0.13 -0.97
```

```
##  Residual             0.9061967 0.95194
## Number of obs: 600, groups:  Week, 4
##
## Fixed effects:
##              Estimate Std. Error        df t value Pr(>|t|)
## (Intercept) -0.001808   0.066539  3.058176  -0.027  0.98000
## RPz          0.170957   0.048029  6.997772   3.559  0.00923 **
## PSSz        -0.237668   0.050715  3.520814  -4.686  0.01274 *
## Anxietyz     0.249142   0.047950  7.157779   5.196  0.00117 **
## ---
## Signif. codes:  0 '***' 0.001 '**' 0.01 '*' 0.05 '.' 0.1 ' ' 1
##
## Correlation of Fixed Effects:
##          (Intr) RPz    PSSz
## RPz      -0.378
## PSSz      0.118 -0.259
## Anxietyz -0.031 -0.134 -0.498
## optimizer (nloptwrap) convergence code: 0 (OK)
## boundary (singular) fit: see ?isSingular
```

Table 3: Depression Included

	Estimate	Std. Error	df	t value	Pr(>|t|)	CI_lower	CI_upper
(Intercept)	-0.007	0.070	3.080	-0.095	0.930	-0.144	0.130
RPz	0.169	0.050	5.591	3.375	0.017	0.071	0.267
PSSz	-0.146	0.085	3.288	-1.717	0.176	-0.312	0.021
Anxietyz	0.305	0.055	9.925	5.524	0.000	0.197	0.413
Depressionz	-0.172	0.088	3.581	-1.944	0.132	-0.345	0.001

Table 4: Depression Removed

	Estimate	Std. Error	df	t value	Pr(>|t|)	CI_lower	CI_upper
(Intercept)	-0.002	0.067	3.058	-0.027	0.980	-0.132	0.129
RPz	0.171	0.048	6.998	3.559	0.009	0.077	0.265
PSSz	-0.238	0.051	3.521	-4.686	0.013	-0.337	-0.138
Anxietyz	0.249	0.048	7.158	5.196	0.001	0.155	0.343

```
m3_NoDepression <- lmer(SRAz ~ RPz + PSSz + Anxietyz + agez + Employment +
             (1+RPz+PSSz+Anxietyz + Employment|Week), data=CVA_full)
```

```
## Linear mixed model fit by REML. t-tests use Satterthwaite's method [
## lmerModLmerTest]
## Formula: SRAz ~ RPz + PSSz + Anxietyz + agez + Employment + (1 + RPz +
##     PSSz + Anxietyz + Employment | Week)
##    Data: CVA_full
##
## REML criterion at convergence: 1588.3
##
## Scaled residuals:
##     Min      1Q  Median      3Q     Max
```

```
## -2.6466 -0.6204 -0.0692  0.6076  3.4487
##
## Random effects:
##  Groups   Name                Variance  Std.Dev. Corr
##  Week     (Intercept)         0.0077857 0.08824
##           RPz                 0.0033829 0.05816  -1.00
##           PSSz                0.0004835 0.02199   0.36 -0.36
##           Anxietyz            0.0015233 0.03903  -0.53  0.53 -0.98
##           EmploymentStudent   0.0124927 0.11177  -0.99  0.99 -0.48  0.64
##           EmploymentUnemployed 0.0531446 0.23053   0.83 -0.83  0.82 -0.92 -0.89
##  Residual                     0.7878001 0.88758
## Number of obs: 600, groups:  Week, 4
##
## Fixed effects:
##                       Estimate Std. Error        df t value Pr(>|t|)
## (Intercept)            0.12332    0.06588   3.79588   1.872  0.13837
## RPz                    0.15716    0.04930   4.92105   3.188  0.02488 *
## PSSz                  -0.15169    0.04561  20.66048  -3.326  0.00326 **
## Anxietyz               0.25263    0.04712   8.17128   5.361  0.00063 ***
## agez                   0.32513    0.04197 579.86529   7.746 4.26e-14 ***
## EmploymentStudent     -0.11111    0.11938   8.71278  -0.931  0.37709
## EmploymentUnemployed  -0.43065    0.14647   3.33134  -2.940  0.05315 .
## ---
## Signif. codes:  0 '***' 0.001 '**' 0.01 '*' 0.05 '.' 0.1 ' ' 1
##
## Correlation of Fixed Effects:
##             (Intr) RPz    PSSz   Anxtyz agez   EmplyS
## RPz         -0.398
## PSSz         0.125 -0.234
## Anxietyz    -0.164 -0.009 -0.471
## agez        -0.070 -0.045  0.146  0.044
## EmplymntStd -0.640  0.275 -0.086  0.174  0.352
## EmplymntUnm  0.191 -0.384  0.053 -0.295 -0.102 -0.223
## optimizer (nloptwrap) convergence code: 0 (OK)
## boundary (singular) fit: see ?isSingular
```

Removing depression from the full model also did not change the significance of the anxiety coefficient, and again made the PSS coefficient statistically significant in the negative direction. Note: Say something about how PSS and anxiety have opposing effects on altruism, and that it is improtant to keep Depression in the model because it and PSS are correlated.

Table 5: Depression Removed

	Estimate	Std. Error	df	t value	Pr(>|t|)	CI_lower	CI_upper
(Intercept)	0.123	0.066	3.796	1.872	0.138	-0.006	0.252
RPz	0.157	0.049	4.921	3.188	0.025	0.061	0.254
PSSz	-0.152	0.046	20.660	-3.326	0.003	-0.241	-0.062
Anxietyz	0.253	0.047	8.171	5.361	0.001	0.160	0.345
agez	0.325	0.042	579.865	7.746	0.000	0.243	0.407
EmploymentStudent	-0.111	0.119	8.713	-0.931	0.377	-0.345	0.123
EmploymentUnemployed	-0.431	0.146	3.331	-2.940	0.053	-0.718	-0.144

Table 6: Depression Included

	Estimate	Std. Error	df	t value	Pr(>|t|)	CI_lower	CI_upper
(Intercept)	0.119	0.070	3.600	1.700	0.172	-0.018	0.256
RPz	0.156	0.051	4.690	3.085	0.030	0.057	0.255
PSSz	-0.084	0.063	5.329	-1.321	0.240	-0.208	0.040
Anxietyz	0.296	0.052	9.249	5.637	0.000	0.193	0.399
Depressionz	-0.133	0.069	5.164	-1.919	0.111	-0.268	0.003
agez	0.314	0.042	577.048	7.489	0.000	0.232	0.396
EmploymentStudent	-0.120	0.119	8.833	-1.012	0.338	-0.353	0.113
EmploymentUnemployed	-0.416	0.149	3.273	-2.784	0.062	-0.709	-0.123

Using Stress instead of PSS

Using the model with only the main predictors, replacing PSS with Stress does not change the direction or significance of the Anxiety predictor.

```
CVA_full$Stressz <- scale(CVA_full$Stress, center = TRUE, scale = TRUE)

m1_Stress <- lmer(SRAz ~ RPz + Stressz + Anxietyz + Depressionz +
             (1+RPz+Stressz+Anxietyz+Depressionz|Week), data=CVA_full)
```

```
## Linear mixed model fit by REML. t-tests use Satterthwaite's method [
## lmerModLmerTest]
## Formula: SRAz ~ RPz + Stressz + Anxietyz + Depressionz + (1 + RPz + Stressz +
##     Anxietyz + Depressionz | Week)
##    Data: CVA_full
##
## REML criterion at convergence: 1662.5
##
## Scaled residuals:
##     Min      1Q  Median      3Q     Max
## -2.8480 -0.6678 -0.0695  0.5979  3.3855
##
## Random effects:
##  Groups   Name        Variance Std.Dev. Corr
##  Week     (Intercept) 0.012776 0.11303
##           RPz         0.002401 0.04900  -1.00
##           Stressz     0.021220 0.14567  -0.26  0.26
##           Anxietyz    0.007949 0.08916   0.09 -0.09 -0.98
##           Depressionz 0.008632 0.09291   0.70 -0.70 -0.87  0.77
##  Residual             0.899831 0.94859
## Number of obs: 600, groups:  Week, 4
##
## Fixed effects:
##              Estimate Std. Error        df t value Pr(>|t|)
## (Intercept) -0.004449   0.068558  3.055038  -0.065   0.9523
## RPz          0.152730   0.048717  6.143817   3.135   0.0196 *
## Stressz      0.007949   0.097207  3.145439   0.082   0.9398
## Anxietyz     0.292267   0.073520  4.126710   3.975   0.0155 *
## Depressionz -0.265704   0.073627  4.410688  -3.609   0.0191 *
## ---
```

```
## Signif. codes:  0 '***' 0.001 '**' 0.01 '*' 0.05 '.' 0.1 ' ' 1
##
## Correlation of Fixed Effects:
##             (Intr) RPz    Strssz Anxtyz
## RPz         -0.419
## Stressz     -0.162  0.013
## Anxietyz     0.043 -0.086 -0.700
## Depressionz  0.366 -0.256 -0.646  0.144
## optimizer (nloptwrap) convergence code: 0 (OK)
## boundary (singular) fit: see ?isSingular
```

Table 7: PSS

	Estimate	Std. Error	df	t value	Pr(>|t|)	CI_lower	CI_upper
(Intercept)	-0.007	0.070	3.080	-0.095	0.930	-0.144	0.130
RPz	0.169	0.050	5.591	3.375	0.017	0.071	0.267
PSSz	-0.146	0.085	3.288	-1.717	0.176	-0.312	0.021
Anxietyz	0.305	0.055	9.925	5.524	0.000	0.197	0.413
Depressionz	-0.172	0.088	3.581	-1.944	0.132	-0.345	0.001

Table 8: Stress

	Estimate	Std. Error	df	t value	Pr(>|t|)	CI_lower	CI_upper
(Intercept)	-0.004	0.069	3.055	-0.065	0.952	-0.139	0.130
RPz	0.153	0.049	6.144	3.135	0.020	0.057	0.248
Stressz	0.008	0.097	3.145	0.082	0.940	-0.183	0.198
Anxietyz	0.292	0.074	4.127	3.975	0.015	0.148	0.436
Depressionz	-0.266	0.074	4.411	-3.609	0.019	-0.410	-0.121

Finally, with respect to the full model, using the Stress scale in place of the PSS did not change the direction of significance of the anxiety coefficient.

```
m3_Stress <- lmer(SRAz ~ RPz + Stressz + Anxietyz + Depressionz + agez + Employment +
             (1+RPz+Stressz+Anxietyz+Depressionz + Employment|Week), data=CVA_full)
```

```
## Linear mixed model fit by REML. t-tests use Satterthwaite's method [
## lmerModLmerTest]
## Formula: SRAz ~ RPz + Stressz + Anxietyz + Depressionz + agez + Employment +
##     (1 + RPz + Stressz + Anxietyz + Depressionz + Employment |          Week)
##    Data: CVA_full
##
## REML criterion at convergence: 1587.3
##
## Scaled residuals:
##     Min      1Q  Median      3Q     Max
## -2.6655 -0.6361 -0.0576  0.6352  3.4551
##
## Random effects:
##  Groups   Name                 Variance Std.Dev. Corr
##  Week     (Intercept)          0.010846 0.10414
```

```
##           RPz                  0.004172 0.06459  -1.00
##           Stressz              0.014720 0.12133  -0.63  0.63
##           Anxietyz             0.008335 0.09129   0.31 -0.31 -0.93
##           Depressionz          0.006311 0.07944   0.78 -0.78 -0.98  0.83
##           EmploymentStudent    0.016798 0.12961  -1.00  1.00  0.63 -0.31 -0.79
##           EmploymentUnemployed 0.067790 0.26037   0.42 -0.42  0.44 -0.74 -0.24
##  Residual                      0.779489 0.88289
##
##
##
##
##
##
##
##  -0.41
##
## Number of obs: 600, groups:  Week, 4
##
## Fixed effects:
##                       Estimate Std. Error        df t value Pr(>|t|)
## (Intercept)            0.12323    0.07125   3.42268   1.729   0.1707
## RPz                    0.14904    0.05089   4.56992   2.929   0.0364 *
## Stressz                0.01283    0.08550   3.48549   0.150   0.8890
## Anxietyz               0.28068    0.07114   4.00637   3.946   0.0168 *
## Depressionz           -0.18743    0.06685   4.79362  -2.804   0.0397 *
## agez                   0.32119    0.04188 584.16281   7.669 7.28e-14 ***
## EmploymentStudent     -0.12047    0.12374   7.24510  -0.974   0.3617
## EmploymentUnemployed  -0.43433    0.15796   3.21762  -2.750   0.0654 .
## ---
## Signif. codes:  0 '***' 0.001 '**' 0.01 '*' 0.05 '.' 0.1 ' ' 1
##
## Correlation of Fixed Effects:
##             (Intr) RPz    Strssz Anxtyz Dprssn agez   EmplyS
## RPz         -0.457
## Stressz     -0.350  0.205
## Anxietyz     0.150 -0.182 -0.685
## Depressionz  0.379 -0.327 -0.666  0.171
## agez        -0.068 -0.038  0.035 -0.011  0.093
## EmplymntStd -0.674  0.319  0.295 -0.115 -0.272  0.346
## EmplymntUnm  0.046 -0.229  0.264 -0.389 -0.168 -0.090 -0.086
## optimizer (nloptwrap) convergence code: 0 (OK)
## boundary (singular) fit: see ?isSingular
```

Table 9: PSS

	Estimate	Std. Error	df	t value	Pr(>|t|)	CI_lower	CI_upper
(Intercept)	0.119	0.070	3.600	1.700	0.172	-0.018	0.256
RPz	0.156	0.051	4.690	3.085	0.030	0.057	0.255
PSSz	-0.084	0.063	5.329	-1.321	0.240	-0.208	0.040
Anxietyz	0.296	0.052	9.249	5.637	0.000	0.193	0.399
Depressionz	-0.133	0.069	5.164	-1.919	0.111	-0.268	0.003
agez	0.314	0.042	577.048	7.489	0.000	0.232	0.396
EmploymentStudent	-0.120	0.119	8.833	-1.012	0.338	-0.353	0.113

	Estimate	Std. Error	df	t value	Pr(>|t|)	CI_lower	CI_upper
EmploymentUnemployed	-0.416	0.149	3.273	-2.784	0.062	-0.709	-0.123

Table 10: Stress

	Estimate	Std. Error	df	t value	Pr(>|t|)	CI_lower	CI_upper
(Intercept)	0.123	0.071	3.423	1.729	0.171	-0.016	0.263
RPz	0.149	0.051	4.570	2.929	0.036	0.049	0.249
Stressz	0.013	0.086	3.485	0.150	0.889	-0.155	0.180
Anxietyz	0.281	0.071	4.006	3.946	0.017	0.141	0.420
Depressionz	-0.187	0.067	4.794	-2.804	0.040	-0.318	-0.056
agez	0.321	0.042	584.163	7.669	0.000	0.239	0.403
EmploymentStudent	-0.120	0.124	7.245	-0.974	0.362	-0.363	0.122
EmploymentUnemployed	-0.434	0.158	3.218	-2.750	0.065	-0.744	-0.125

Donations with and without depression

Table 11: Depression

	Estimate	Std. Error	df	t value	Pr(>|t|)	CI_lower	CI_upper
(Intercept)	0.109	0.074	3.184	1.464	0.234	-0.037	0.254
RPz	0.130	0.050	4.783	2.586	0.051	0.032	0.229
Anxietyz	0.213	0.059	5.442	3.621	0.013	0.098	0.328
Depressionz	-0.153	0.065	12.811	-2.377	0.034	-0.280	-0.027
PSSz	-0.029	0.067	5.300	-0.433	0.682	-0.161	0.103
agez	0.283	0.053	3.951	5.337	0.006	0.179	0.387
EmploymentStudent	-0.036	0.126	6.673	-0.289	0.781	-0.283	0.210
EmploymentUnemployed	-0.445	0.150	3.271	-2.959	0.053	-0.739	-0.150

Table 12: No Depression

	Estimate	Std. Error	df	t value	Pr(>|t|)	CI_lower	CI_upper
(Intercept)	0.110	0.076	3.263	1.444	0.237	-0.039	0.259
RPz	0.131	0.052	4.792	2.530	0.055	0.029	0.232
Anxietyz	0.157	0.056	4.671	2.811	0.040	0.048	0.267
PSSz	-0.110	0.054	6.094	-2.042	0.086	-0.216	-0.004
agez	0.294	0.059	3.456	4.998	0.011	0.178	0.409
EmploymentStudent	-0.025	0.130	5.829	-0.190	0.856	-0.280	0.231
EmploymentUnemployed	-0.451	0.149	3.384	-3.026	0.048	-0.742	-0.159

Addressing model singularity (revisions August 2022)

To address the singular fit in our main models, we sequentially removed random effects until the model was no longer singular. The ?isSingular command in lmer explains that there is no consensus on how best to deal with singularity, but provides a number of empirically justified ways to deal with it. We opted for Barr

et al.'s (2013) "keep it maximal" approach. We considered the model in its current singular state to be our maximal model (random slopes for all coefficients and a random intercept) and began taking random effects out until it was no longer singular. This resulted in a model with only random intercept per week.

```
m1.1 <- lmer(SRAz ~ RPz + PSSz + Anxietyz + Depressionz +
             (1|Week), data=CVA_full)
```

```
## Linear mixed model fit by REML. t-tests use Satterthwaite's method [
## lmerModLmerTest]
## Formula: SRAz ~ RPz + PSSz + Anxietyz + Depressionz + (1 | Week)
##    Data: CVA_full
##
## REML criterion at convergence: 1659.1
##
## Scaled residuals:
##     Min      1Q  Median      3Q     Max
## -2.8194 -0.6605 -0.0785  0.5682  3.3990
##
## Random effects:
##  Groups   Name        Variance Std.Dev.
##  Week     (Intercept) 0.01195  0.1093
##  Residual             0.89918  0.9483
## Number of obs: 600, groups:  Week, 4
##
## Fixed effects:
##               Estimate Std. Error         df t value Pr(>|t|)
## (Intercept) -3.249e-04  6.698e-02  2.996e+00  -0.005   0.9964
## RPz          1.681e-01  4.230e-02  5.950e+02   3.975 7.91e-05 ***
## PSSz        -1.461e-01  5.698e-02  5.944e+02  -2.563   0.0106 *
## Anxietyz     3.117e-01  5.109e-02  5.931e+02   6.101 1.90e-09 ***
## Depressionz -1.688e-01  6.250e-02  5.943e+02  -2.702   0.0071 **
## ---
## Signif. codes:  0 '***' 0.001 '**' 0.01 '*' 0.05 '.' 0.1 ' ' 1
##
## Correlation of Fixed Effects:
##             (Intr) RPz    PSSz   Anxtyz
## RPz          0.000
## PSSz         0.000 -0.200
## Anxietyz     0.000 -0.165 -0.054
## Depressionz  0.000  0.012 -0.585 -0.451
```

Table 13: Table m1.1

	Estimate	Std. Error	df	t value	Pr(>|t|)	CI_lower	CI_upper
(Intercept)	0.000	0.067	2.996	-0.005	0.996	-0.132	0.131
RPz	0.168	0.042	594.973	3.975	0.000	0.085	0.251
PSSz	-0.146	0.057	594.443	-2.563	0.011	-0.258	-0.034
Anxietyz	0.312	0.051	593.092	6.101	0.000	0.212	0.412
Depressionz	-0.169	0.062	594.289	-2.702	0.007	-0.291	-0.046

```r
m3.1 <- lmer(SRAz ~ RPz + PSSz + Anxietyz + Depressionz + agez + Employment +
             (1|Week), data=CVA_full)
```

```
## Linear mixed model fit by REML. t-tests use Satterthwaite's method [
## lmerModLmerTest]
## Formula: SRAz ~ RPz + PSSz + Anxietyz + Depressionz + agez + Employment +
##     (1 | Week)
##    Data: CVA_full
##
## REML criterion at convergence: 1596
##
## Scaled residuals:
##     Min      1Q  Median      3Q     Max
## -2.6911 -0.6226 -0.0251  0.5821  3.4251
##
## Random effects:
##  Groups   Name        Variance Std.Dev.
##  Week     (Intercept) 0.01501  0.1225
##  Residual             0.79764  0.8931
## Number of obs: 600, groups:  Week, 4
##
## Fixed effects:
##                         Estimate Std. Error        df t value Pr(>|t|)
## (Intercept)              0.12476    0.07858   4.41271   1.588 0.180900
## RPz                      0.14759    0.03995 591.59589   3.694 0.000241 ***
## PSSz                    -0.08806    0.05422 590.86101  -1.624 0.104898
## Anxietyz                 0.30646    0.04817 589.86119   6.362 4.01e-10 ***
## Depressionz             -0.12883    0.05911 590.78564  -2.179 0.029697 *
## agez                     0.30814    0.04215 591.24845   7.310 8.73e-13 ***
## EmploymentStudent       -0.12682    0.10612 589.03532  -1.195 0.232536
## EmploymentUnemployed    -0.40699    0.09062 590.82055  -4.491 8.52e-06 ***
## ---
## Signif. codes:  0 '***' 0.001 '**' 0.01 '*' 0.05 '.' 0.1 ' ' 1
##
## Correlation of Fixed Effects:
##             (Intr) RPz    PSSz   Anxtyz Dprssn agez   EmplyS
## RPz         -0.001
## PSSz         0.047 -0.203
## Anxietyz    -0.015 -0.164 -0.056
## Depressionz  0.000  0.006 -0.569 -0.447
## agez        -0.056 -0.060  0.069  0.012  0.087
## EmplymntStd -0.313 -0.001 -0.050  0.044  0.033  0.397
## EmplymntUnm -0.337  0.005 -0.120  0.013 -0.031 -0.162  0.198
```

Table 14: Table m3.1

	Estimate	Std. Error	df	t value	Pr(>|t|)	CI_lower	CI_upper
(Intercept)	0.125	0.079	4.413	1.588	0.181	-0.029	0.279
RPz	0.148	0.040	591.596	3.694	0.000	0.069	0.226
PSSz	-0.088	0.054	590.861	-1.624	0.105	-0.194	0.018
Anxietyz	0.306	0.048	589.861	6.362	0.000	0.212	0.401
Depressionz	-0.129	0.059	590.786	-2.179	0.030	-0.245	-0.013

	Estimate	Std. Error	df	t value	Pr(>|t|)	CI_lower	CI_upper
agez	0.308	0.042	591.248	7.310	0.000	0.226	0.391
EmploymentStudent	-0.127	0.106	589.035	-1.195	0.233	-0.335	0.081
EmploymentUnemployed	-0.407	0.091	590.821	-4.491	0.000	-0.585	-0.229
